# Sertoli cell-only phenotype and scRNA-seq define PRAMEF12 as a factor essential for spermatogenesis in mice

**DOI:** 10.1038/s41467-019-13193-3

**Published:** 2019-11-15

**Authors:** Zhengpin Wang, Xiaojiang Xu, Jian-Liang Li, Cameron Palmer, Dragan Maric, Jurrien Dean

**Affiliations:** 1 Laboratory of Cellular and Developmental Biology, NIDDK, National Institutes of Health, Bethesda, MD 20892 USA; 2 Integrative Bioinformatics, NIEHS, National Institutes of Health, Research Triangle Park, NC 27709 USA; 3 NINDS Flow Cytometry Core Facility, National Institutes of Health, Bethesda, MD 20892 USA

**Keywords:** Spermatogenesis, Stem cells

## Abstract

Spermatogonial stem cells (SSCs) have the dual capacity to self-renew and differentiate into progenitor spermatogonia that develop into mature spermatozoa. Here, we document that preferentially expressed antigen of melanoma family member 12 (PRAMEF12) plays a key role in maintenance of the spermatogenic lineage. In male mice, genetic ablation of *Pramef12* arrests spermatogenesis and results in sterility which can be rescued by transgenic expression of *Pramef12*. *Pramef12* deficiency globally decreases expression of spermatogenic-related genes, and single-cell transcriptional analysis of post-natal male germline cells identifies four spermatogonial states. In the absence of *Pramef12* expression, there are fewer spermatogonial stem cells which exhibit lower expression of SSC maintenance-related genes and are defective in their ability to differentiate. The disruption of the first wave of spermatogenesis in juvenile mice results in agametic seminiferous tubules. These observations mimic a Sertoli cell-only syndrome in humans and may have translational implications for reproductive medicine.

## Abstract (web summary)

Spermatogonial stem cells have the dual capacity to self-renew and differentiate into mature spermatozoa. Here, using transcriptome analyses of juvenile testes in gene-edited mice, the authors demonstrate that PRAMEF12 is required to maintain germ cell homeostasis and promote their differentiation.

## Introduction

**Fig. 1:**
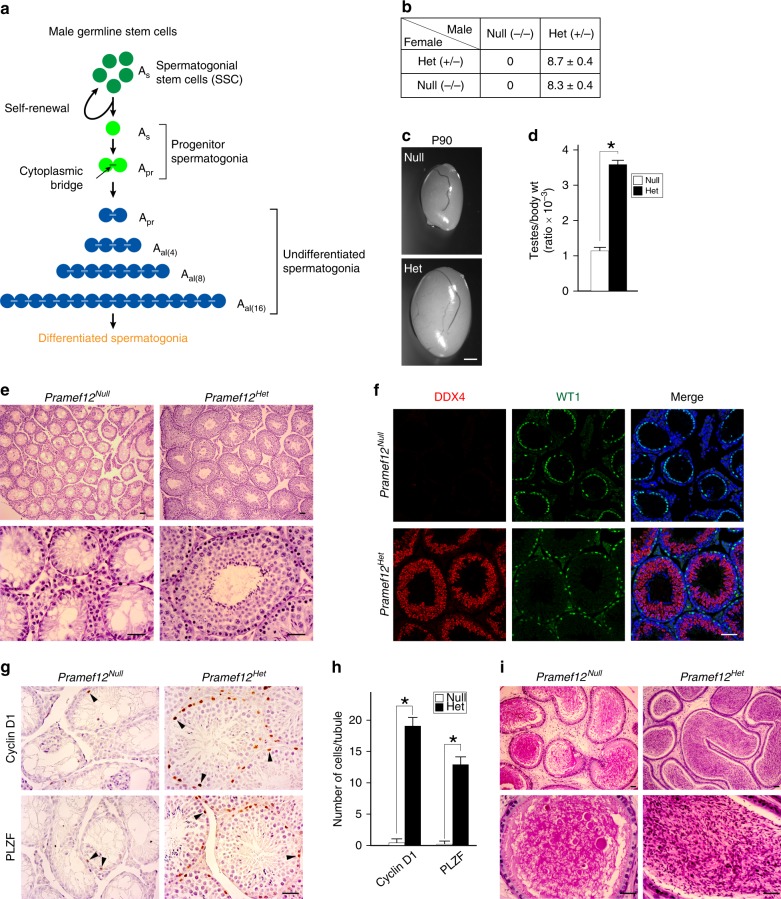
*Pramef12* is essential for spermatogenesis and male fertility. **a** The hierarchy of different cell types of spermatogonia during SSC self-renewal and differentiation. The A_s_ spermatogonia are heterogeneous with SSCs and A_s_ progenitors. The A_s_ and A_pr_ progenitors have the potential to become SSCs. A_s_ spermatogonia produce chains of A_pr_ A_al(4)_, A_al(8)_, and A_al(16)_ undifferentiated spermatogonia that are connected by cytoplasmic bridges and are precursors of differentiated spermatogonia. **b** Fertility of > 3 pairs of *Pramef12*^*Null*^ and *Pramef12*^*Het*^ male and female mice mated 1:1. Mean litter sizes ± s.d. are shown with indicated genotypes. **c** Testes of P90 adult *Pramef12*^*Null*^ and *Pramef12*^*Het*^ mice. Scale bar, 1 mm. **d** Ratios of testis to body weight of *Pramef12*^*Null*^ and *Pramef12*^*Het*^ mice shown in **c**. Mean ± s.d, *n* = 6 biologically independent testes from six different animals. *P *=* 1.44E-08 by two-tailed Student’s *t* test. **e** Adult testis sections from *Pramef12*^*Null*^ and *Pramef12*^*Het*^ mice stained with periodic acid-Schiff (PAS) and hematoxylin. *Pramef12*^*Null*^ testes are agametic with a Sertoli cell-only phenotype. Scale bar, 50 μm. **f** Immunofluorescence of P90 adult testes from *Pramef12*^*Null*^ and *Pramef12*^*Het*^ mice after co-staining with antibodies to DDX4 (germ cells) and WT1 (Sertoli cells) as well as Hoechst 33342 (DNA). Scale bar, 50 μm. **g** Immunohistochemistry of P90 adult testes from *Pramef12*^*Null*^ and *Pramef12*^*Het*^ mice after staining with antibodies to cyclin D1 and PLZF. Arrowheads indicate positive-staining spermatogonia. Scale bar, 50 μm. **h** Quantification of cyclin D1-positive and PLZF-positive spermatogonia in *Pramef12*^*Null*^ and *Pramef12*^*Het*^ testes. Mean ± s.d, *n* = 3 biologically independent testes from three different animals. *P *=* 1.44E-28 (cyclin D1) and *P *=* 3.19E-26 (PLZF) by two-tailed Student’s *t* test. **i** Same as **e**, but of cauda epididymides. Representative of *n* = 6 **c**, *n* = 3 **e**, **f**, **g**, **i** independent biological replicates with similar results per condition

Mammalian spermatogenesis is a highly coordinated, dynamic process of cell differentiation with three distinc phases: mitois, meiosis, and spermiogenesis. Life-long testicular spermatogenesis depends on the presence of spermatogonial stem cells (SSCs) as well as their amplification and transition into progenitor and differentiating spermatogonia^1,2^. In mice, SSCs are derived from gonocytes that arise from primordial germ cells (PGCs) formed in the proximal epiblast during embryogenesis^3–6^. SSCs are located in the basal compartment of the seminiferous epithelium of the testis and have the capacity to divide which: (1) allows self-renewal to maintain the SSC pool; and (2) gives rise to progenitor spermatogonia, which have a large proliferative capacity, but are committed to differentiate^7^. SSCs reside in the population of A_single_ (A_s_) spermatogonia, which initiate mitotic divisions either to produce two new A_s_ spermatogonia by complete cytokinesis or to generate chains of A_paired_ (A_pr_), and A_aligned_ (A_al(4)_, A_al(8)_, A_al(16)_, and in rare cases, A_al(32)_) spermatogonia (Fig. 1a). Because of incomplete cytokinesis, A_pr_ and A_al_ cells are connected by intercellular bridges and represent undifferentiated spermatogonia^2,4,6^. The A_al_ spermatogonia, as well as a few A_pr_ spermatogonia, differentiate into A_1_ spermatogonia without division and then mitotically divide five times to sequentially form A_2_, A_3_, A_4_, intermediate, and type B spermatogonia that are collectively termed differentiated spermatogonia^1,2,4^. Type B spermatogonia divide into two primary diploid spermatocytes, each of which meiotically divide into four haploid round spermatids to initiate spermiogenesis and form mature spermatozoa^2^.

A_s_ SSCs represent 0.02–0.03% of all germ cells in the adult testes^8^. At present, three models for SSC renewal have been proposed^9,10^. In the classic A_single_ model, the daughters of a dividing A_s_ cell either contribute to maintenance of the SSC pool or begin to differentiate. In the fragmentation model, disintegration of larger clones (i.e., A_aligned_) into single and pairs of cells significantly contribute to the maintenance of the SSC pool^11–15^. More recently, a hierarchical model proposes that restricted expression of ID4, PAX7, and/or BMI1 define a subset of spermatogonia as authentic stem cells^9,10,16,17^. Of note, few SSC marker proteins are specific to male germ cells and at present, mouse SSCs cannot be defined by the presence of a specific stem cell protein(s).

FIGLA (factor in the germline, alpha) is a germline specific basic helix-loop-helix transcription factor required for mouse oocyte differentiation. Although expressed in male germ cells, *Figla*^*Null*^ male mice appear normal and are fertile^18^. In a screen for downstream gene targets in the mouse ovary, PRAMEF12, a member of a PRAME multigene family, was identified^19^. Preferentially expressed antigen of melanoma (PRAME) was first discovered in human melanoma cell lines, and is a tumor-associated antigen recognized by cytolytic T lymphocytes^20^. The *PRAME* family genes, although present in humans and other mammals, are absent in zebrafish, amphibians, and invertebrates, indicating that the *PRAME* gene family is eutheria-specific^21^. The PRAME protein family belongs to a group of cancer-testis antigens that are aberrantly expressed in a variety of cancers and, in normal adult tissues, restricted to the testis and ovary^22,23^. Members of the PRAME gene family encode leucine-rich repeats, a structural motif frequently involved in protein–protein interactions^24,25^. It has been reported that PRAME family proteins function as transcription regulators in cancer cells and may play roles in spermatogenesis and oogenesis^26,27^. *PRAME* genes can be separated into groups according to their expression pattern in mice: testis (*Prame, Pramel1, Pramel 3*)^26,28^, ovary (*Oogenesin1-4*)^29–31^, embryo (*Pramel4-7*)^32,33^, and both male and female gonads (*Pramel, Pramef8, Pramef12*)^26^. However, the function of PRAME proteins remains unclear and no knockout mice have been reported. Using gene-edited mice, we now report the essential role of *Pramef12* in maintaining SSC homeostasis and facilitating germ cell differentiation to ensure male fertility.

## Results

### Spermatogonial loss and infertility in *Pramef12*^*Null*^ mice

To investigate the function of *Pramef12* in germ cell development, we established PRAMEF12 null mice using CRISPR/Cas9. Two founder lines lacking either 37 or 49 bp after the start codon were obtained and bred to homozygosity (Supplementary Fig. 1a–d). *Pramef12*^*−/−*^ female mice had normal fertility with litters the same size as *Pramef12*^*+/−*^ females when bred with *Pramef12*^*+/−*^ male mice. In contrast, *Pramef12*^*−/−*^ males were sterile and produced no pups when co-caged with either *Pramef12*^*+/−*^ or *Pramef12*^*−/−*^ female mice (Fig. 1b). *Pramef12*^*−/−*^ males from each mutant line exhibited similar characteristics of testicular hypoplasia (Supplementary Fig. 1e, f), and subsequent studies were focused on the line containing a 37 bp deletion, which we refer to as *Pramef12*^*Null*^ and use heterozygous null mice from the same line (designated *Pramef12*^*Het*^) as controls.

Testes isolated from 3-month-old adult *Pramef12*^*Null*^ mice were significantly smaller and weighed less than controls (Fig. 1c, d). Histologically, mutant seminiferous tubules were agametic and contained only somatic cells that resembled the Sertoli cell-only syndrome associated with human infertility (Fig. 1e). The absence of germ cells was confirmed by immunofluorescence (IF) in which the germ cell-specific marker DEAD box polypeptide 4 (DDX4) was not detected, but Wilms tumor 1 (WT1), a Sertoli cell-specific marker, was observed (Fig. 1f). Immunohistochemical analyses using antibodies for cyclin D1 (a marker for mitotically active spermatogonia) and promyelocytic leukemia zinc finger, PLZF (official name Zbtb16, a marker for undifferentiated spermatogonia) in control and *Pramef12*^*Null*^ testes indicate that cyclin D1-positive and PLZF-positive spermatogonia were rarely detected and their numbers were remarkably reduced in mutant testes (Fig. 1g, h). No sperm were present in the cauda epididymides isolated from *Pramef12*^*Null*^ mice (Fig. 1i) which, taken together, indicates that PRAMEF12 is essential for spermatogenesis.

**Fig. 2:**
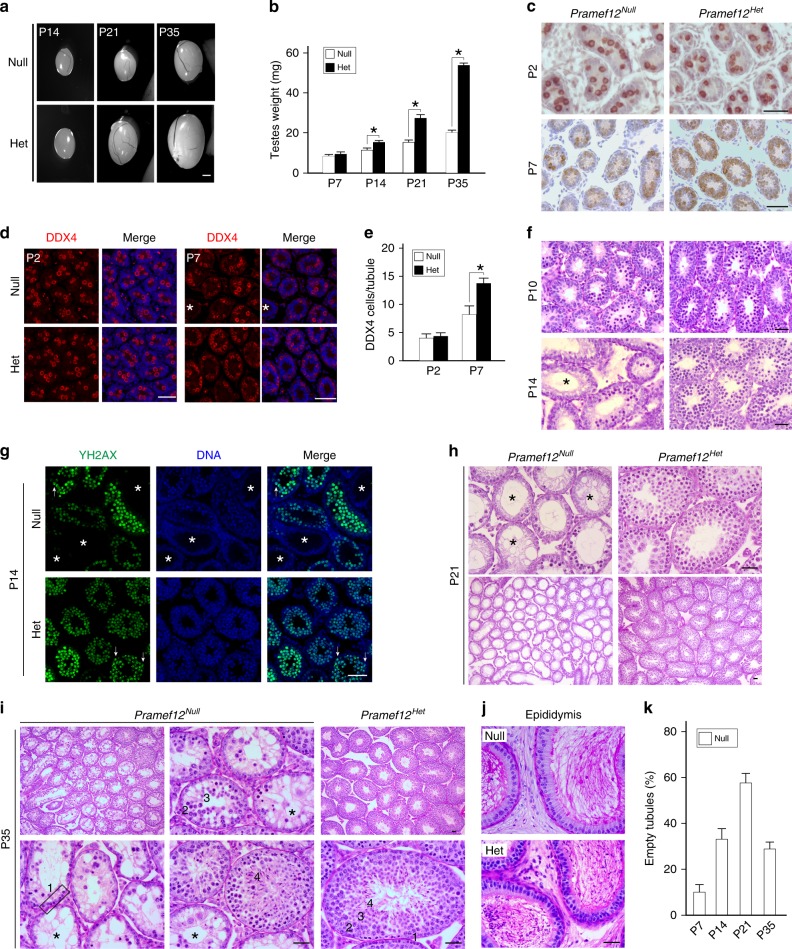
*Pramef12* is required for the first wave of spermatogenesis. **a** Morphology of testes from P14, P21, and P35 *Pramef12*^*Null*^ and *Pramef12*^*Het*^ mice. Scale bar, 1 mm. **b** Comparison of testes weight of P7, P14, P21, and P35 *Pramef12*^*Null*^ and *Pramef12*^*Het*^ mice. Mean ± s.d, *n* = 5 biologically independent testes from five different animals. *P *=* 3.81E-03 (P14), *P *=* 2.38E-06 (P21) and *P *=* 4.84E-08 (P35) by two-tailed Student’s *t* test. Immunohistochemistry **c** and immunofluorescence **d** staining of DDX4 in the testicular sections from P2 and P7 *Pramef12*^*Null*^ and *Pramef12*^*Het*^ mice. The merge in **d** is with Hoechst 33342 stained DNA. Scale bar, 50 μm. **e** Quantification of DDX4-positive cells per tubules in *Pramef12*^*Null*^ and *Pramef12*^*Het*^ testes at P2 and P7. Mean ± s.d, *n* = 3 biologically independent testes from three different animals. *P *=* 2.71E-07 by two-tailed Student’s *t* test. **f** Testicular sections of P10 and P14 *Pramef12*^*Null*^ and *Pramef12*^*Het*^ mice were stained with periodic acid-Schiff (PAS) and hematoxylin. Scale bar, 50 μm. **g** Immunofluorescence staining of γH2AX in testicular sections from P14 *Pramef12*^*Null*^ and *Pramef12*^*Het*^ mice. DNA was stained with Hoechst 33342. Arrows, pachytene spermatocytes (XY body); asterisks, Sertoli cell-only tubules. Scale bar, 50 μm. **h**, **i** PAS staining of P21 and P35 testes in *Pramef12*^*Null*^ and *Pramef12*^*Het*^ mice. The reduction in germ cell number becomes notable with the appearance of more agametic tubules (asterisks). Spermatogonia, 1; spermatocytes, 2; spermatids, 3; and elongated spermatozoa, 4; asterisks, agametic tubules. Scale bar, 50 μm. **j** Same as **i**, but of cauda epididymides. **k** Percentage of agametic tubules in *Pramef12*^*Null*^ testes from P7 to P35. *Pramef12*^*Het*^ control testes contain no empty tubules. Mean ± s.d, *n* = 3 biologically independent testes from 3 different animals at each age point. Representative of *n* = 5 **a**, *n* = 3 **c**, **d**, **f**–**j** independent biological replicates with similar results per condition

To determine the stage at which defects in spermatogenesis occurred, testes size and weight from control and mutant mice were compared from post-natal day 7 (P7) to P35 (Fig. 2a, b). Histologic and immunohistochemical analyses with DDX4 staining document that mutant testes had normal numbers of spermatogonia at P2. However, spermatogonia were either completely depleted or significantly reduced in mutant seminiferous tubules by P7 (Fig. 2c–e), revealing a defect in the proliferative expansion of spermatogonia that normally occurs during this period. To determine whether mutant spermatogonia could enter meiosis, P10 and P14 testicular sections were stained with PAS and P14 sections were stained with antibodies to γH2AX. Mutant testes contained γH2AX-positive spermatocytes although in diminished numbers, suggesting that mutant spermatogonia could enter meiosis (Fig. 2f, g). From P14 to P35, testes from *Pramef12*^*Null*^ mice were significantly smaller in size and lower in weight than controls (Fig. 2a, b). At P14, some mutant tubules were either devoid of germ cells or contained diminished numbers of spermatogenic cells (Fig. 2f, g). These defects were more severe at P21, in which more than half of the *Pramef12*^*Null*^ tubules were agametic and the remaining tubules contained reduced numbers of germ cells (Fig. 2h). At P35, when the first wave of spermatogenesis is complete, elongated spermatids and mature sperm were present in control testicular tubules and cauda epididymides. However, the mutant testes exhibited similar phenotypes as at P21 with few or no spermatogenic cells in seminiferous tubules, which contained numerous vacuoles (Fig. 2i, j). No spermatozoa were present in mutant cauda epididymides despite of the presence of round and elongating spermatids in a few tubules (Fig. 2i, j). Empty tubules depleted of germ cells were observed at P7, reached a maximum level at P21 and decreased at P35 in *Pramef12*^*Null*^ testes (Fig. 2k). The spermatogenic cell loss was further confirmed by dual immunostaining with antibodies to DDX4 and WT1 in control and mutant developing testes from P14 to P35 (Supplementary Fig. 2a). Terminal deoxynucleotidyl transferase dUTP nick end labeling (TUNEL) analysis exhibited a significant increase in apoptosis within mutant seminiferous tubules at P14 and P35 (Supplementary Fig. 2b, d). However, the number of Sertoli cells in mutant testes did not differ from control testes (Supplementary Fig. 2a, c).

**Fig. 3:**
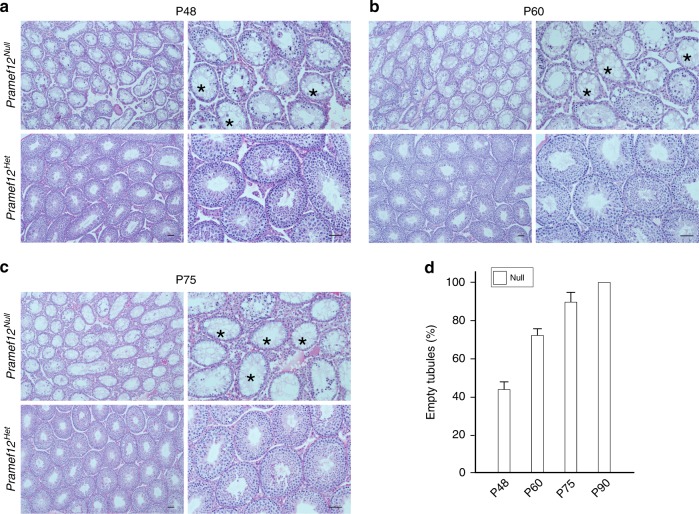
Further evaluation of spermatogenic phenotype of *Pramef12*^*Null*^ mice. **a** Testicular sections of P48 *Pramef12*^*Null*^ and *Pramef12*^*Het*^ mice were stained with periodic acid-Schiff (PAS) and hematoxylin. Asterisks, agametic tubules. Scale bar, 50 μm. **b** Same as **a**, but of P60 testes. **c** Same as **a**, but of P75 testes. **d** Statistical analysis of empty tubules from P48 to P90 *Pramef12*^*Null*^ mice. Mean ± s.d, *n* = 3 biologically independent testes from three different animals at each age point. Representative of *n* = 3 **a**–**c** independent biological replicates with similar results per condition

To determine whether the sterility of P90 *Pramef12*^*Null*^ males arose from a lack of germline cells or a stage-specific block in spermatogenic differentiation, we extended our investigations of the *Pramef12*^*Null*^ phenotype from P48 to P75. Histological analyses documented that the defects in spermatogenesis were more severe in mutant testes which contained more agametic tubules and markedly diminished spermatogenesis from P48 to P75 compared with P35 *Pramef12*^*Null*^ testes (Fig. 2i, Fig. 3a–c). Statistical analysis indicated that there was a significant age-dependent increase in the number of empty tubules from P35 to P90 (Fig. 2k, Fig. 3d) that substantiates the conclusion that the sterility at P90 is owing to the absence of germ cells. Taken together, these observations indicate that *Pramef12* deficiency causes a dramatic loss of spermatogonia and spermatogenic cells that disrupts the first wave of spermatogenesis in juvenile testes. This results in agametic seminiferous tubules in adult testes and male infertility.

### PRAMEF12 expression in spermatogonial cells

**Fig. 4:**
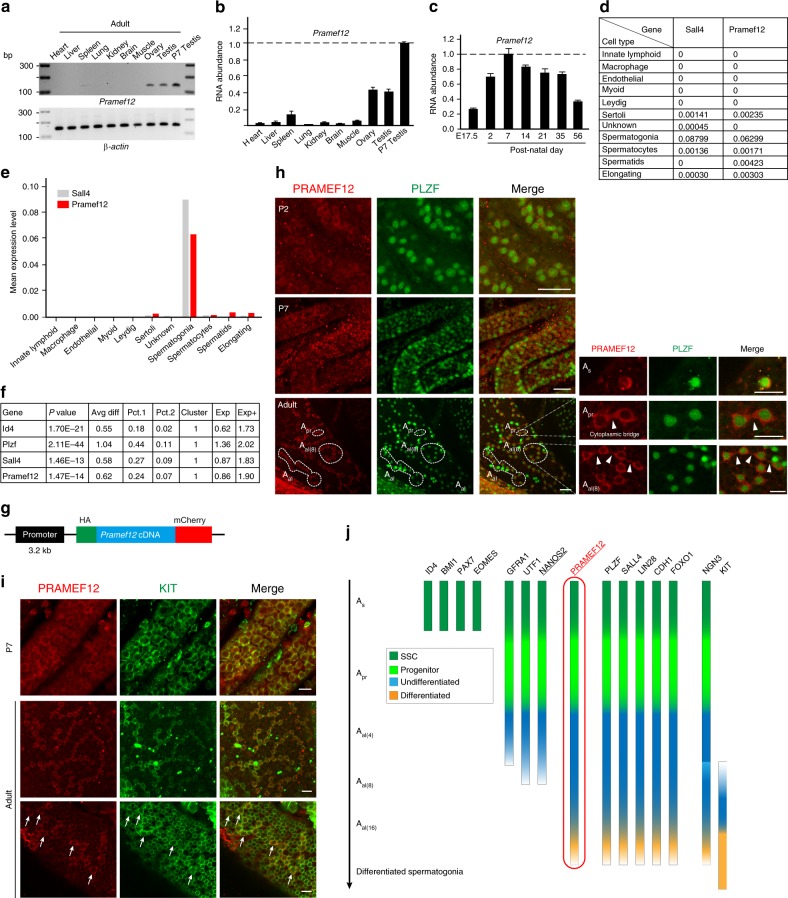
PRAMEF12 is specifically expressed in spermatogonia. **a**–**c** Abundance of *Pramef12* transcripts in different adult tissues and P7 testes **a**, **b** and E17.5-P56 testes **c** was determined by RT-PCR **a** or qRT-PCR **b**, **c** using *β-actin* as a control. Molecular mass, left in **a**. The highest expression level of *Pramef12* relative to *β-actin* was set to 1 in **b**, **c**. Mean ± s.d, *n* = 3 biologically independent samples per condition. **d**–**f** Analyses of the abundance of *Pramef12* transcripts in 11 testicular cell types **d**, **e** and in cluster 1 of re-clustered germ cells **f** based on previously reported scRNA-seq data in adult mouse testes^34^. Numbers in **d** and the *Y* axis in **e** reflect the averaged UMIs detected in each individual cell. After re-clustering germ cells, *Id4*, *Plzf,* and *Sall4* serve as positive controls and, like *Sall4*, *Pramef12* was primarily enriched in the spermatogonial cluster 1, which corresponds to undifferentiated spermatogonia **f**. *P* value in **f** was obtained from the published scRNA-seq data^34^. Avg diff, log_2_-scale fold change. Percent of cells expressing gene in cluster 1 (Pct.1) or in non-cluster 1 cells (Pct.2). Exp and Exp + reflect mean expression of the marker in all cells of this cluster and in the marker-positive cells of this cluster, respectively. **g** Schematic representation of transgene expressing ^HA^*Pramef12*^mCherry^ cDNA driven by the *Pramef12* promoter (3.2 kb). **h** Whole-mount staining of P2, P7 and adult *Pramef12*^*HA/mCherry*^ testes with antibodies to mCherry (left), PLZF (middle) and merged (right). Scale bar, 50 μm. Examples of A_s_, A_pr_, and A_al_ undifferentiated spermatogonia in adult testes (dotted circles) and enlarged (right panels). Arrowheads, high-light cytoplasmic location. Scale bar, 20 μm. **i** Same as **h** for P7 and adult testes, but with antibodies to mCherry (left), KIT (middle) and merged (right). Arrows, high-light co-localization of the two markers. **j** The expression profile of intracellular molecular markers for male germline cells. Except for NANOS2 (underlined), PRAMEF12 (underlined, red) is unique in its cell type specificity and is largely enriched in undifferentiated spermatogonia. Representative of *n* = 3 **a**, **h**, **i** independent biological replicates with similar results per condition

RT-PCR documented that *Pramef12* transcripts were largely restricted to the ovary and testis and were most abundant in P7 testes (Fig. 4a–c). Using recently reported scRNA-seq data from adult mouse testis^34^, PRAMEF12 transcripts are not detected in adult Leydig, myoid, endothelial, macrophage cells, and barely detected in Sertoli cells. In contrast, PRAMEF12 is highly expressed in spermatogonia (~ 27-fold higher than in Sertoli cells) (Fig. 4d, e). Moreover, PRAMEF12 transcripts are mainly expressed in spermatogonial subtype 1 (cluster 1), which corresponds to undifferentiated spermatogonia across the identified four spermatogonial subtypes in the adult mouse testis (Fig. 4f). Our own scRNA-seq analysis of P7 mice confirmed these observations as will be described later in Results.

To localize the protein in the absence of a commercially available antibody, we established *Pramef12*^*HA/mCherry*^ transgenic mouse line expressing *Pramef12* cDNA tagged with HA and mCherry at the N- and C-termini, respectively (Fig. 4g). Using a 3.2 kb *Pramef12* promoter to drive expression of the recombinant protein and whole-mounts of P2, P7, and adult transgenic testes, cellular localization of PRAMEF12 was determined by confocal microscopy (Fig. 4h, i). To define the identity and classification of PRAMEF12-positive cells, we co-stained PRAMEF12 with PLZF, which is a marker of undifferentiated spermatogonia, although it is also expressed in early stages of differentiation^35^. Immunostaining indicated that PRAMEF12 was expressed in the cytoplasm of spermatogonia and co-stained with PLZF in most undifferentiated spermatogonia at P2 and P7. Co-expression of PRAMEF12 and PLZF suggested that PRAMEF12 was possibly expressed in some A_s_, A_pr_, and chains of A_al_ undifferentiated spermatogonia of adult *Pramef12*^*HA/mCherry*^ mouse testis (Fig. 4h). A small fraction of undifferentiated spermatogonia were positive for PLZF without an mCherry signal (Fig. 4h), indicating that the expression pattern of PRAMEF12 is consistent with molecular heterogeneity among undifferentiated spermatogonia^14^.

To investigate expression of PRAMEF12 in spermatogonia poised to differentiate, we co-stained whole-mount testes with antibodies to KIT, a marker for differentiated spermatogonia. Co-expression of PRAMEF12 and KIT was detected in P7 seminiferous tubules (Fig. 4i). PRAMEF12 and KIT also co-stained chained A_al_ spermatogonia, but the abundance of PRAMEF12 was downregulated as spermatogonia further differentiated in adult *Pramef12*^*HA/mCherry*^ testis (Fig. 4i). These observations suggest that PRAMEF12 persists and is co-expressed with KIT in clones of A_al_ spermatogonia which are possibly in transition from undifferentiated to differentiating spermatogonia. In summary, PRAMEF12 is primarily enriched in undifferentiated spermatogonia, but is also present in some early KIT-positive differentiating spermatogonia in mouse testis (Fig. 4j).

### PRAMEF12 is required for stem cell maintenance

**Fig. 5:**
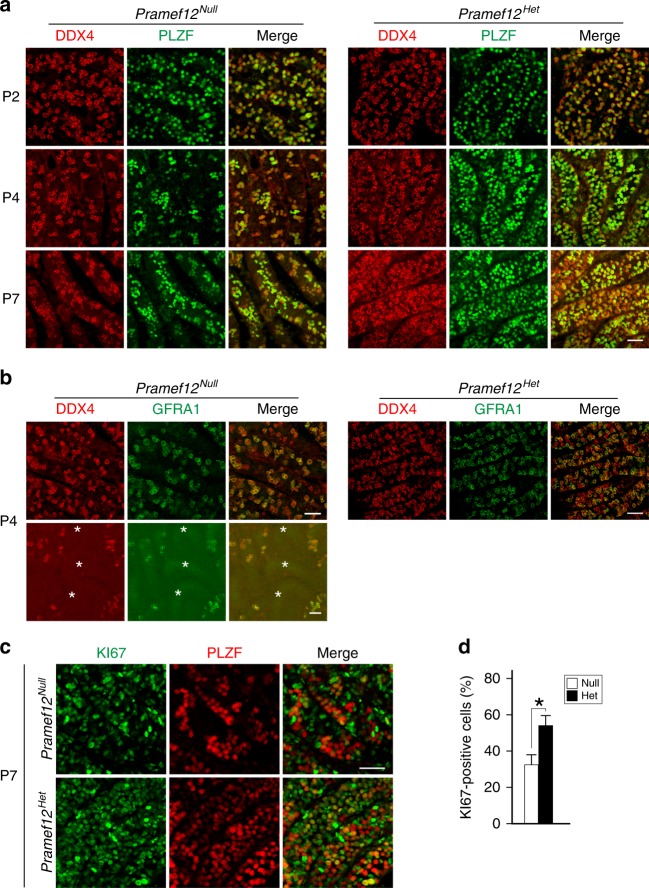
Essential role of PRAMEF12 in early spermatogenic maintenance. **a** Immunofluorescence of whole-mount testes from P2, P4, and P7 *Pramef12*^*Null*^ (left group) and *Pramef12*^*Het*^ (right group) mice after staining with antibodies to DDX4 (left) or PLZF (middle) and merged (right). The number of PLZF-positive spermatogonia was comparable in P2 *Pramef12*^*Null*^ and *Pramef12*^*Het*^ tubules and significantly decreased in *Pramef12*^*Null*^ tubules as early as at P4, although still present at P7. Scale bar, 50 μm. **b** Same as **a**, but with antibodies to GFRA1 (co-receptor for GDNF) at P4. Asterisks, agametic tubules. **c** Same as **a**, but with antibodies to KI67 (marker of mitosis) and PLZF using whole-mount testes from *Pramef12*^*Null*^ (top) and *Pramef12*^*Het*^ (bottom) mice at P7. **d** The percent of KI67^+^; PLZF^+^ double-positive among the total PLZF^+^ single-positive spermatogonia in P7 *Pramef12*^*Null*^ and *Pramef12*^*Het*^ tubules. At least six random regions of individual testis were counted from at least three different mice. Mean ± s.d, *n* = 3 biologically independent samples per condition. *P *=* 5.89E-23 by two-tailed Student’s *t* test. Representative of *n* = 3 **a**–**c** independent biological replicates with similar results per condition

Because gonocytes re-enter mitosis shortly after birth, we determined the overall number of germ cells and undifferentiated spermatogonia from P2, P4, and P7 tubules after whole-mount staining with antibodies to DDX4 and PLZF. The number of DDX4-positive germ cells and PLZF-positive spermatogonia were comparable in control and *Pramef12*^*Null*^ tubules at P2. However, the number was substantially reduced in mutant tubules by P4 and P7 (Fig. 5a), which was confirmed by morphometric analyses after staining with antibodies to PLZF (Supplementary Fig. 3a, d). The number of GFRA1-positive spermatogonia was significantly decreased in mutant tubules as early as P4 at which time tubules without DDX4-positive germ cells were observed (Fig. 5b). TUNEL assays indicated that the number of apoptotic spermatogonia in mutant testes was not significantly different from controls (Supplementary Fig. 3e, f), suggesting that these defects were not primarily due to increased cell death.

Compared with controls, the average number of cyclin D1- and PCNA- (marker of proliferation) positive spermatogonia per tubule cross-section was significantly lower in mutant testes (Supplementary Fig. 3b–d). Whole-mount co-staining for PLZF and KI67 (a marker for mitotic spermatogonia) in P7 seminiferous tubules also demonstrated that the number of PLZF^+^KI67^+^ spermatogonia was dramatically reduced in mutant tubules (Fig. 5c, d). Immunoblots documented that expression of DDX4, PLZF, LIN28A (a marker for undifferentiated spermatogonia), and cyclin D1 was significantly decreased in P7 mutant testes compared with controls (Supplementary Fig. 3g). Thus, the proliferative capacity of SSCs is impaired in *Pramef12*^*Null*^ mice, which leads to a defect in spermatogonial expansion that normally occurs in early post-natal testes. We also observed a significant and rapid reduction in the overall number of PLZF-positive and cyclin D1-positive spermatogonia in mutant testes from P14 to P35 mice (Supplementary Fig. 4a–d). Overall, these results suggest that PRAMEF12 is required for SSC self-renewal and proliferation, and that disruption of *Pramef12* impairs early spermatogenic maintenance and causes age-related decline of undifferentiated spermatogonial populations.

### PRAMEF12 is essential for spermatogonial differentiation

**Fig. 6:**
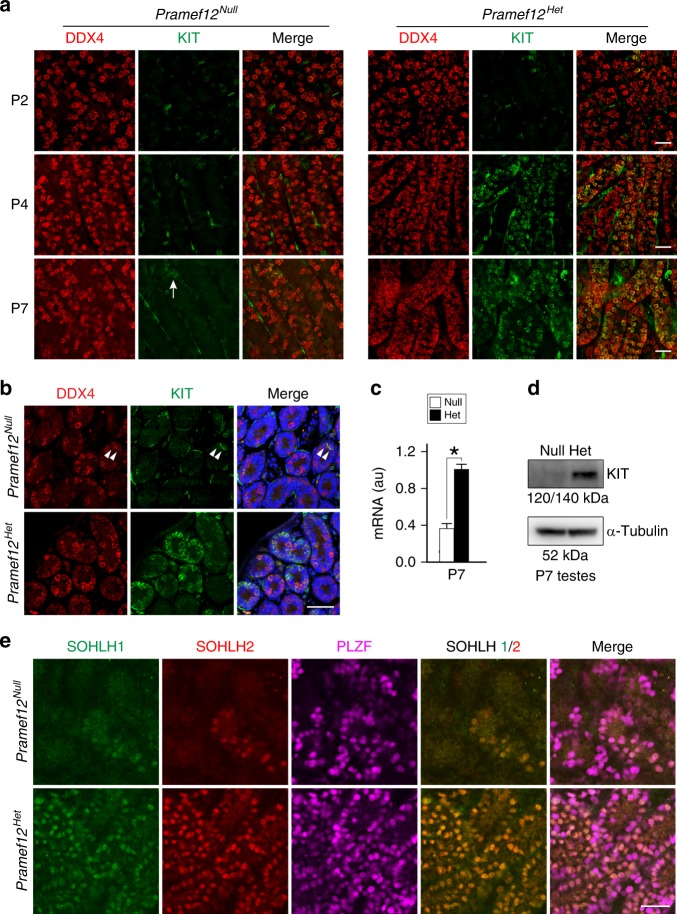
Essential role of PRAMEF12 in spermatogonial differentiation. **a** Immunofluorescence of whole-mount testes from P2, P4, and P7 *Pramef12*^*Null*^ and *Pramef12*^*Het*^ mice after staining with antibodies to DDX4 (left) or KIT (middle) and merged (right). KIT-positive spermatogonia were rarely detected at P2 in either *Pramef12*^*Null*^ or *Pramef12*^*Het*^ tubules but were present at P4 and increased at P7 in *Pramef12*^*Het*^ tubules. Few KIT-positive spermatogonia (arrow) were observed in P7 *Pramef12*^*Null*^ tubules. Scale bar, 50 μm. **b** Immunohistochemistry of sections from P7 *Pramef12*^*Null*^ (top panels) and *Pramef12*^*Het*^ (bottom panels) testes after staining with antibodies to DDX4 (left) or KIT (middle) and merged with Hoechst 33342 to stain DNA (right). Arrowheads, KIT-positive cells in *Pramef12*^*Null*^ testes. Scale bar, 50 μm. **c** Quantitative real-time RT-PCR of *Kit* mRNA abundance in P7 *Pramef12*^*Null*^ and *Pramef12*^*Het*^ testes using *β-actin* as an internal load control and setting the abundance in *Pramef12*^*Het*^ testis to 1. Mean ± s.d, *n* = 3 biologically independent samples per condition. *P *=* 1.43E-05 by two-tailed Student’s *t* test. **d** Immunoblot of KIT protein in P7 *Pramef12*^*Null*^ and *Pramef12*^*Het*^ testes using α-tubulin as a load control. **e** Same as **a**, but with antibodies to SOHLH1, SOHLH2, and PLZF. The number of spermatogonia positive for SOHLH1 and SOHLH2 was significantly reduced in P7 *Pramef12*^*Null*^ tubules (top panels). Representative of *n* = 3 **a**, **b**, **d**, **e** independent biological replicates with similar results per condition

To investigate the role of PRAMEF12 in spermatogonial differentiation, we determined that KIT-positive spermatogonia in both control and mutant seminiferous tubules were rarely detected at P2. However, the number of KIT-positive spermatogonia in mutant tubules was greatly diminished as early as P4 when a small portion of KIT-positive spermatogonia appeared in the control tubules. This expression pattern persisted and became more notable by P7 (Fig. 6a). A marked reduction in the number of KIT-positive spermatogonia was also observed in sections of mutant testes at P7 (Fig. 6b). These observations were confirmed by real-time RT-PCR and immunoblot, which documented that KIT mRNA and protein abundance was significantly reduced in P7 mutant testes (Fig. 6c, d). Some mutant tubules exhibited spermatogonial arrest and failure of differentiation despite the presence of PLZF- and cyclin D1-positive spermatogonia at the basement membrane as evidenced by the absence of multiple stages of differentiating germ cells (Supplementary Fig. 4a, c). These results suggest that although mutant testes contain a small population of PLZF- or cyclin D1-positive spermatogonia, these cells are unable to differentiate further. SOHLH1 and SOHLH2 have been implicated in coordinating spermatogonial differentiation^36^ and the numbers of SOHLH1- and SOHLH2-positive spermatogonia were markedly decreased in mutant seminiferous tubules compared with controls by P7 (Fig. 6e). Taken together, these data indicate that PRAMEF12 has an important role in spermatogonial differentiation.

### Recombinant PRAMEF12 rescues *Pramef12*^*Null*^ male mice

We sought to use the *Pramef12*^*HA/mCherry*^ transgenic mice to rescue the defects in spermatogenesis and fertility of *Pramef12*^*Null*^ mice. *Pramef12*^*HA/mCherry*^ transgenic mice crossed into *Pramef12*^*Null*^ mice had decreased testicular size and weight compared with either *Pramef12*^*Het*^ or *Pramef12*^*Het*^*;Pramef12*^*HA/mCherry*^ littermates, but were comparable to *Pramef12*^*Null*^ littermates (Supplementary Fig. 5a, b). About 20% of the seminiferous tubules in *Pramef12*^*Null*^*;Pramef12*^*HA/mCherry*^ males contained spermatogenic cells and a few mature sperm were present in the cauda epididymis (Supplementary Fig. 5c, d). Because of the low efficiency of rescue, we generated a second transgenic mouse line in which recombinant PRAMEF12 was tagged with FLAG-6xHis and HA at the N- and C-termini, respectively (Supplementary Fig. 5e). *Pramef12*^*Null*^*;Pramef12*^*FLAG/6xHis/HA*^ rescue males had significantly larger testis size and weight compared with *Pramef12*^*Null*^ littermates, albeit slightly smaller than *Pramef12*^*Het*^ or *Pramef12*^*Het*^*;Pramef12*^*FLAG/6xHis/HA*^ mice (Supplementary Fig. 5f, g). Restoration of all stages of spermatogenesis was observed in *Pramef12*^*Null*^*;Pramef12*^*FLAG/6xHis/HA*^ seminiferous tubules with normal numbers of mature sperm in the cauda epididymides (Supplementary Fig. 5h, i, k). Although the abundance of *Pramef12* and *Eomes* mRNA in the *Pramef12*^*Null*^*;Pramef12*^*FLAG/6xHis/HA*^ testis was only partially restored at P7 (Supplementary Fig. 5j), *Pramef12*^*Null*^*;Pramef12*^*FLAG/6xHis/HA*^ males had normal fertility when mated with *Pramef12*^*Null*^ females (Supplementary Fig. 5k). Overall, these data suggest that recombinant PRAMEF12 can substitute for the endogenous protein to rescue spermatogenesis and fertility in *Pramef12*^*Null*^ mice despite only partial restoration of the protein.

### *Pramef12* deficiency affects spermatogenic gene expression

**Fig. 7:**
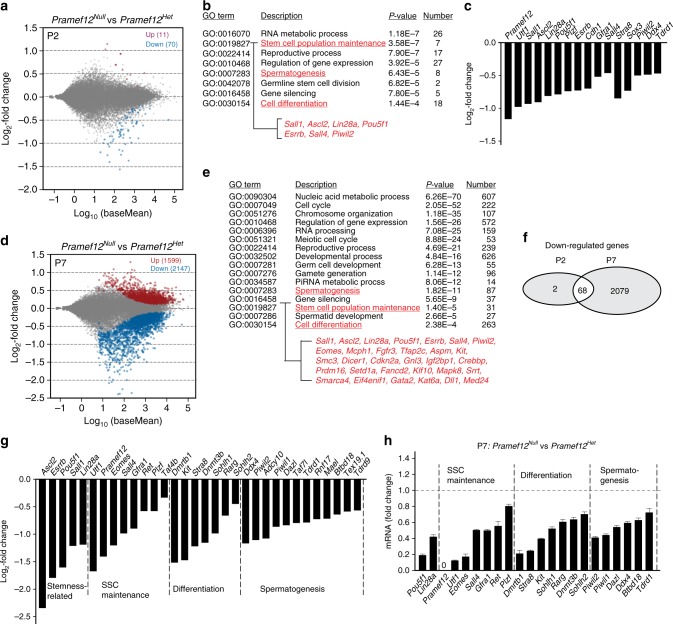
*Pramef12* deficiency alters expression patterns of spermatogenic genes. **a** MA plot (log ratio RNA abundance versus abundance) of RNA-seq data from *Pramef12*^*Null*^ and *Pramef12*^*Het*^ testes at P2. 11 genes and 70 genes were upregulated and downregulated, respectively, in *Pramef12*^*Null*^ testes using adjusted *P* *<* 0.1 as the cut off. **b** Gene ontology of downregulated genes in P2 *Pramef12*^*Null*^ testes. Genes that support stem cell population maintenance indicated in red text fly-out. GO terms were obtained by GOrilla analysis. *P* value was obtained from the data by GOrilla analysis. **c** Genes related to stem cell maintenance and differentiation that were significantly downregulated (log_2_-fold change) in P2 *Pramef12*^*Null*^ testes. **d** Same as **a**, but at P7. In all, 1599 genes and 2147 genes were upregulated and downregulated in *Pramef12*^*Null*^ testes, respectively. **e** Same as **b**, but at P7. *P* value was obtained from the data by GOrilla analysis. **f** Venn diagram depicting the overlap of downregulated genes determined at two developmental time points, P2 and P7. **g** RNA-seq results of selected transcripts (log_2_-fold change) related to SSC maintenance, differentiation and spermatogenesis that were significantly downregulated in P7 *Pramef12*^*Null*^ testes. **h** Quantitative RT-PCR validation of downregulated genes involved in SSC maintenance, differentiation and spermatogenesis in *Pramef12*^*Null*^ testes at P7. For comparison, the abundance (relative to *β-actin*) of each gene in *Pramef12*^*Het*^ control mice was set to 1. Data are presented as mean ± s.d for *n* = 3 biologically independent samples per condition

To investigate the molecular consequences of the loss of *Pramef12*, we compared the transcriptomes of *Pramef12*^*Null*^ and *Pramef12*^*Het*^ testes at P2 and P7. RNA-seq analysis identified 11 upregulated and 70 downregulated genes at P2 using adjusted *P* *<* 0.1 (Fig. 7a). Gene ontology (GO) analysis documented that downregulated transcripts were significantly enriched for those that function in RNA metabolic processes, stem cell population maintenance (e.g., *Sall1*, *Ascl2*, *Lin28a*, *Pou5f1*, *Esrrb*, *Sall4*, *Piwil2*), reproductive process, spermatogenesis (e.g., *Ddx4*, *Adcy10*, *Tdrd1*, *Bnc1*, *Stra8*), gene silencing and cell differentiation (e.g., *Cdh1*, *Foxc2*, *Stra8*, *Sox3*) (Fig. 7b). Some known transcripts (e.g., *Utf1*, *Plzf*, *Gfra1*, *Sall4*, *Foxc2*, *Sox3*) involved in SSC maintenance and differentiation were significantly reduced in P2 mutant testes (Fig. 7c). Using the same criterion, we found that 1599 genes were upregulated and 2147 genes were downregulated in P7 mutant testes (Fig. 7d). Among downregulated genes at the two time points, most of the genes (68/70) downregulated at P2 were also significantly downregulated at P7 and *Pramef12*^*Null*^ testes at P7 showed a substantially higher number of downregulated genes (Fig. 7f). The genes significantly downregulated at P7 included those involved in cell cycle (222 genes), regulation of gene expression (572 genes), RNA processing (159 genes), meiotic cell cycle (53 genes), reproductive process (239 genes), spermatogenesis (87 genes including *Ddx4*, *Adcy10*, *Dazl*, *Taf7l*, *Tdrd1*, *Rnf17*, *Mael*, *Btbd18*), stem cell population maintenance (31 genes including *Ascl2*, *Esrrb*, *Sall1*, *Eomes*, *Utf1*, *Pou5f1*, *Lin28a*, *Sall4*, *Gfra1*, *Ret*, *Plzf*) as well as cell differentiation (263 genes including *Dmrtb1*, *Kit*, *Stra8*, *Dnmt3b*, *Rarg*, *Sohlh1*, *Sohlh2*) (Fig. 7e, g). We next verified the expression patterns of the genes by real-time RT-PCR at P7. The qRT-PCR results confirmed significant downregulation of two stemness-related genes (*Pou5f1*, *Lin28a*), seven genes involved in SSC maintenance (*Pramef12*, *Utf1*, *Eomes*, *Sall4*, *Gfra1*, *Ret*, *Plzf*), seven genes involved in spermatogonial differentiation (*Dmrtb1*, *Stra8*, *Kit*, *Sohlh1*, *Rarg*, *Dnmt3b*, *Sohlh2*) and six genes involved in spermatogenesis (*Piwil2*, *Piwil1*, *Dazl*, *Ddx4*, *Btbd18*, *Tdrd1*) (Fig. 7h). Taken together, these data suggest that *Pramef12* deficiency globally influences the expression of genes that control multiple spermatogenic processes, including SSC maintenance and differentiation.

### scRNA-seq defines the transcriptome of *Pramef12*^*Null*^ testes

**Fig. 8:**
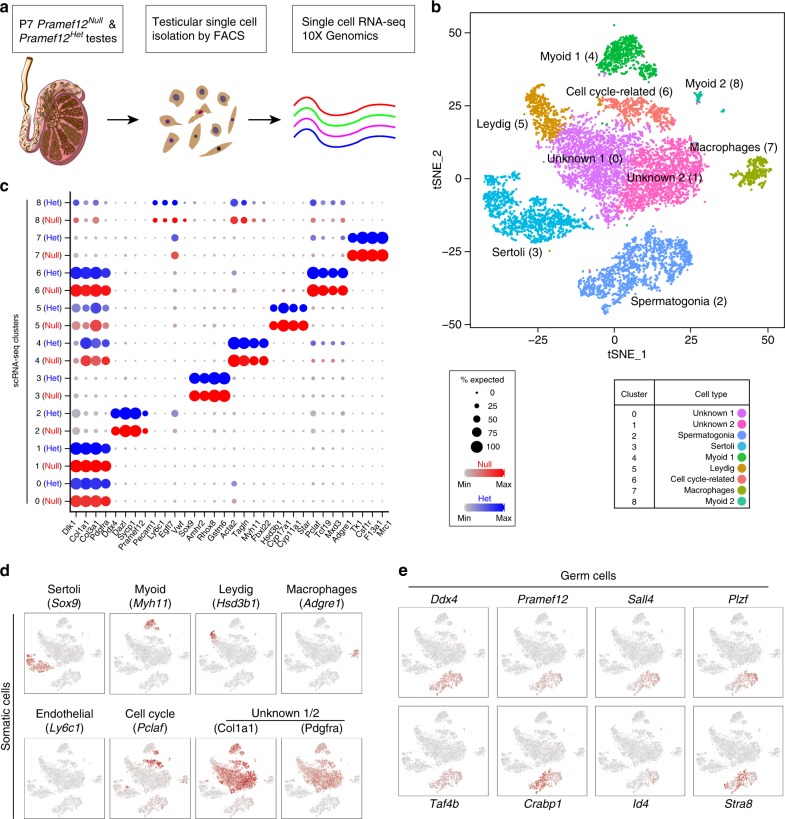
Single-cell transcriptome of *Pramef12*^*Het*^ and *Pramef12*^*Null*^ testicular cells. **a** Schematic illustration of the workflow for scRNA-seq analysis. **b** tSNE and clustering analysis of combined single-cell transcriptome data from P7 *Pramef12*^*Het*^ and *Pramef12*^*Null*^ testicular cells. Each dot represents a single-cell and cell clusters are distinguished by colors. **c** Dotplot for expression of selected marker genes across all identified cell types. **d** Gene expression patterns of selected somatic cell marker genes projected on the tSNE plots. **e** Expression patterns of marker genes for germ cells visualized in tSNE plots. *Pramef12* is exclusively expressed in germ cell population but not in somatic cell populations

To address the cellular heterogeneity of spermatogonia, we isolated single cells from six testes of P7 *Pramef12*^*Het*^ and *Pramef12*^*Null*^ mice, respectively, and performed scRNA-seq analysis using the 10X Genomics platform (Fig. 8a). From a total of 5473 *Pramef12*^*Het*^ and 5116 *Pramef12*^*Null*^ testicular cells, 5221 and 4839 cells passed standard quality control and were retained for subsequent analysis (Supplementary Fig. 6a). On average, we detected 7446 unique molecular indices (UMIs) and 2450 genes in each individual cell, which are sufficient to define distinct cell types in mouse testis.

Unsupervised clustering of the total 10,060 testicular cells projected onto t-distributed stochastic neighbor embedding (tSNE) analysis plots identified nine major cell types (Fig. 8b). None of the clusters consisted of cells derived solely from either *Pramef12*^*Het*^ or *Pramef12*^*Null*^ testes (Supplementary Fig. 6b). Cluster identity was assigned based on expression patterns of known marker genes in mouse testis (Fig. 8c–e, Supplementary Fig. 6c, d, Supplementary Data 1). We identified a germ cell population expressing spermatogonia-specific genes and eight somatic cell populations including five known somatic cell populations: Sertoli, myoid, Leydig, endothelial, and macrophages and three new cell populations: a cell cycle-related cell population and two unknown cell populations expressing fibroblast-specific marker genes including *Col1a1*, *Col3a1*, *Pdgfra,* and *Dlk1*. *Pramef12* was primarily expressed in the germ cell population and was not expressed in somatic cell populations (Fig. 8c, e) consistent with previous findings in adult mouse testis^34^.

### Transcriptome signatures of *Pramef12*^*Null*^ testes

**Fig. 9:**
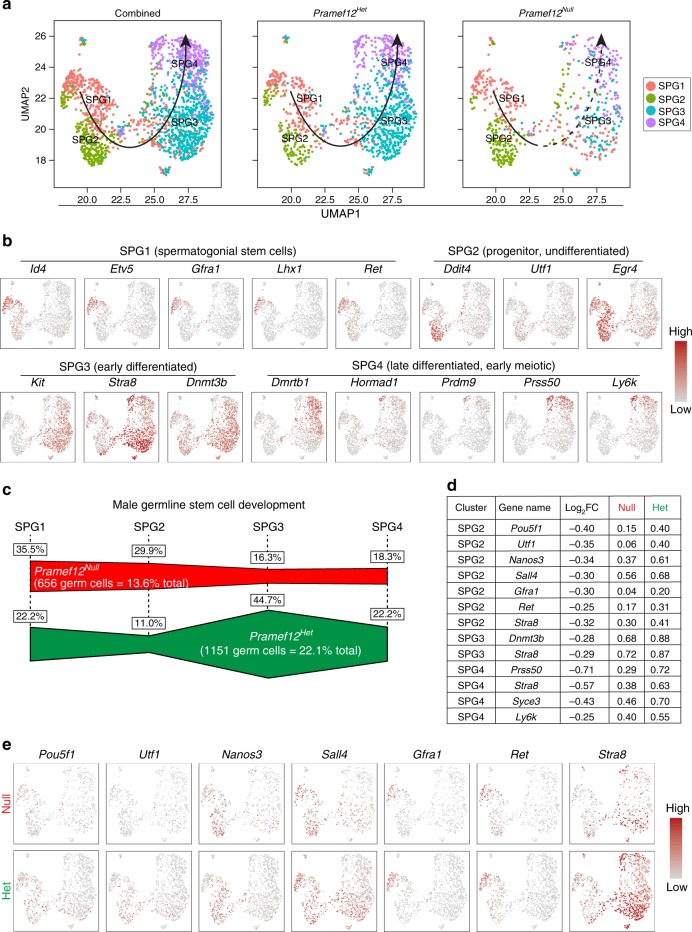
Transcriptome-wide signatures of germline stem cell development. **a** Focused analysis (UMAP clustering and pseudotime ordering) of combined (left), *Pramef12*^*Het*^ (middle), and *Pramef12*^*Null*^ (right) germ cells documented four biological subtypes following the order of SPG1 to SPG4 (state 1 to state 4). **b** Gene expression patterns of selected marker genes corresponding to each cellular state on the UMAP plots. **c** Summary schematic depicting the percentage of spermatogonia in each cellular state in *Pramef12*^*Null*^ and *Pramef12*^*Het*^ testes. *Pramef12* deficiency significantly impairs germline stem cell development as determined by the decreased percentage of differentiated cells in SPG3 and SPG4. **d** Differentially expressed genes (DEGs) between *Pramef12*^*Null*^ and *Pramef12*^*Het*^ cells in SPG2, SPG3, and SPG4. Log_2_FC, log_2_-fold change. Null and Het indicate the percentage of cells in which the gene was detected in *Pramef12*^*Null*^ and *Pramef12*^*Het*^ samples, respectively. **e** UMAP plots of the expression patterns of selected DEGs in SPG2 subtype in *Pramef12*^*Null*^ and *Pramef12*^*Het*^ samples

To determine the transcriptome-wide signatures of SSC development and how PRAMEF12 effects this process, we re-clustered the 1153 *Pramef12*^*Het*^ and 654 *Pramef12*^*Null*^ spermatogonial cells. Uniform Manifold Approximation and Projection (UMAP) and marker gene analyses were performed for cell type identification^37^. Based on UMAP, we observed four distinct spermatogonial subtypes/states. Pseudotime analysis provided an arrow vector, which aligned with the developmental order of germline stem cells from spermatogonial state 1 to state 4 (SPG1 to SPG4) (Fig. 9a). None of the clusters/states derived solely from *Pramef12*^*Het*^ or *Pramef12*^*Null*^ spermatogonial cells (Supplementary Fig. 7a). Differentially expressed genes (DEGs) were observed among the four clusters and several classic spermatogonial marker genes for each state were identified in our single-cell data (Fig. 9b, Supplementary Fig. 7b, Supplementary Data 2). Gene ontology analysis identified the top several terms for these DEGs in each of the SPG1-4 states (Supplementary Fig. 7c). Marker gene analysis suggests that SPG1 cells correspond to SSCs, as they highly express SSC marker genes including *Id4*, *Etv5*, *Gfra1*, *Lhx1,* and *Ret* (Fig. 9b). SPG2 cells express *Ddit4*, *Utf1*, *Egr4*, *Pramef12*, *Sall4,* and *Plzf* and display low expression of differentiation markers (Fig. 9b, Supplementary Fig. 7d) consistent progenitor/undifferentiated spermatogonia. In contrast, SPG3 cells express high levels of early differentiation marker genes such as *Kit*, *Stra8* and *Dnmt3b* and low levels of *Pramef12*, *Sall4* and *Plzf* (Fig. 9b, Supplementary Fig. 7d) which indicates that this cell population is likely early differentiating spermatogonia. SPG4 cells mainly express late differentiation marker genes including *Prss50* and *Ly6k* and high levels of early meiotic genes such as *Hormad1* and *Prdm9* (Fig. 9b), implicating them as differentiated spermatogonia. Therefore, the expression patterns of spermatogonial marker genes recapitulate the developmental order of germline cells from SPG1 to SPG4. In addition, we found that the expression pattern of *Pramef12* is similar to *Sall4*, *Plzf*, *Lin28a* and *Sdc4* in spermatogonia (Supplementary Fig. 7d).

We determined that 22.2%, 11.0%, 44.7%, and 22.2% cells sorted into SPG1, SPG2, SPG3, and SPG4, respectively, in *Pramef12*^*Het*^ sample and 35.5%, 29.9%, 16.3%, and 18.3% cells sorted into SPG1, SPG2, SPG3, and SPG4, respectively, in *Pramef12*^*Null*^ sample (Fig. 9c). A dramatic decrease in the percentage of SPG3 and SPG4 cells in the *Pramef12*^*Null*^ sample suggests that *Pramef12* deficiency severely impairs germline stem cell development. Identification and analysis of differentially expressed genes between *Pramef12*^*Null*^ and *Pramef12*^*Het*^ cells in SPG subtypes revealed that *Utf1*, *Nanos3*, *Sall4*, *Gfra1,* and *Ret*, required in maintaining the undifferentiated spermatogonial population, and *Stra8*, important for spermatogonial differentiation, were significantly reduced in mutant SPG2 cells (Fig. 9d, Supplementary Data 3). Consistently, genes related to spermatogonial differentiation such as *Stra8* and *Dnmt3b*, were markedly downregulated in mutant SPG3 cells. Although *Prss50*, *Stra8*, *Syce3*, *Syce2*, *Tex101*, *Rhox13,* and *Ly6k* were largely decreased in mutant SPG4 cells (Fig. 9d, Supplementary Data 3). These results further demonstrate that *Pramef12*-deficient spermatogonia exhibit lower expression of several SSC maintenance- and differentiation-related genes. Moreover, differential expression analysis of the expression patterns of above genes further documented that the percentage of cells expressing several SSC maintenance-related genes in SPG2 in *Pramef12*^*Null*^ cells was sharply reduced and cells expressing differentiation marker genes in SPG3 and SPG4 were significantly downregulated compared with *Pramef12*^*Het*^ cells (Fig. 9d, e, Supplementary Fig. 7e). Taken together, single-cell transcriptional analysis of male germline cells indicates that PRAMEF12 is crucial for germline stem cell maintenance and differentiation.

### SSC fate in *Pramef12*^*Null*^ mice

**Fig. 10:**
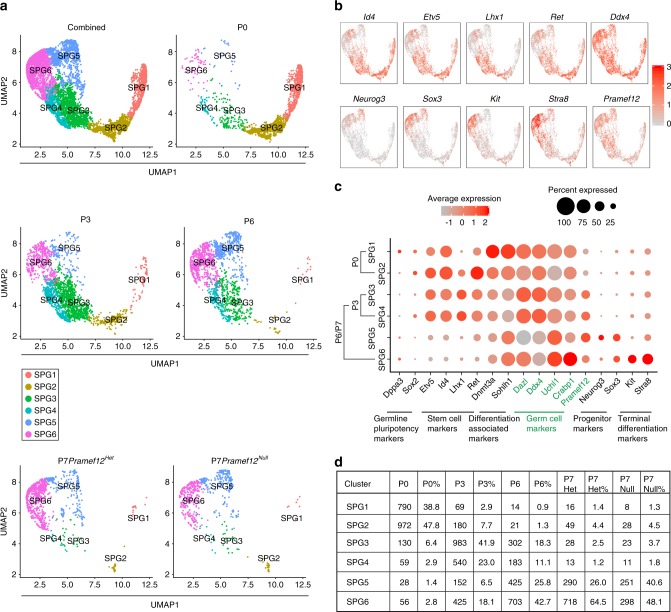
Spermatogonial stem cell fates in *Pramef12*^*Null*^ male mice. **a** UMAP clustering of the combined germ cells including P0, P3, P6, P7 *Pramef12*^*Het*^, and *Pramef12*^*Null*^ samples documented six biological subtypes following a developmental trajectory of SPG1 to SPG6 (upper left). Five additional UMAP plots specific to each individual sample (right and below). **b** Gene expression patterns of selected spermatogonial marker genes corresponding to each cellular state on the UMAP plots: SPG1 (*Id4*, *Etv5*, *Ret*); SPG2 (*Id4*, *Etv5*, *Lhx1*, *Ret*); SPG3 (*Id4*, *Etv5*, *Lhx1*, *Ret*); SPG4 (*Id4*, *Etv5*, *Lhx1*, *Ret*); SPG5 (*Neurog3*, *Sox3*); and SPG6 (*Kit*, *Stra8*). **c** Dotplot representation of average scaled expression and the percentage of cells within each cluster for selected marker genes across all ages and cell types. **d** Table of the distribution and the percentage of the cells within each cluster and each sample

A recent study documents the developmental kinetics and transcriptome dynamics of SSC specification in the spermatogenic lineage^38^. To define the SSC fate in the absence of PRAMEF12, we combined the published scRNA-seq data on germ cells isolated from P0, P3, and P6 testes^38^ with our scRNA-seq data on P7 *Pramef12*^*Het*^ and *Pramef12*^*Null*^ germ cells, and re-clustered the spermatogonial cells. We observed six distinct spermatogonial subtypes/states based on UMAP and differential gene expression (Fig. 10a). The identity of each state was determined by the age distribution of cells in each cluster and spermatogonial marker genes (Fig. 10a–d). P0 cells (86.6%) were mainly enriched in SPG1 and SPG2, which expressed high levels of SSC markers *Id4*, *Etv5*, *Ret,* and moderate levels of *Lhx1*. P3 cells (64.9%) were primarily sorted into SPG3 and SPG4 that possessed high levels of SSC-associated markers including *Id4*, *Etv5*, *Ret*, and *Lhx1*. 10.6% cells clustered into SPG1 and SPG2, and 24.6% cells clustered into SPG5 and SPG6 corresponding to differentiated spermatogonia. P6, P7 *Pramef12*^*Het*^, and P7 *Pramef12*^*Null*^ cells shared some overlap among the clusters and were primarily represented by clusters 3-6. Cluster 5 possesed low levels of SSC-associated markers, high levels of progenitor markers (*Neurog3*, *Sox3*) and low levels of terminal differentiation markers (*Kit*, *Stra8*), identifying them as progenitor spermatogonia formed from the SSC pool. Cluster 6 possesed the lowest levels of SSC-associated markers and the highest expression of terminal differentiation markers (*Kit*, *Stra8*), which characterized them as more differentiated spermatogonia. Based on the above analyses of known markers, the developmental trajectory of SSCs is from SPG1 to SPG6. Interestingly, the distribution of cells in cluster 5 in P7 *Pramef12*^*Null*^ cells is much higher than in P0 (40.6% versus 1.4%), in P3 (40.6% versus 6.5%), in P6 (40.6% versus 25.8%), and in P7 *Pramef12*^*Het*^ (40.6% versus 26.0%) cells (Fig. 10d), suggesting that a higher percentage of progenitor spermatogonia were present in *Pramef12*^*Null*^ testes. This indicates that in the absence of PRAMEF12, the SSCs had lower stem cell potential and more closely resembled progenitor spermatogonia, which is supported by the transcriptome analyses in which a higher percentage of cells remained as progenitor spermatogonia. This implies that *Pramef12* deficiency significantly impairs stem cell potential and blocks stem cell differentiation at an early stage of spermatogenesis consistent with our findings (Fig. 9) that *Pramef12* deficiency impedes germline stem cell development.

## Discussion

Mammalian spermatogenesis is a dynamic process that is sustained by a balance between maintaining a population of self-renewing SSCs and the ability of these cells to differentiate into mature spermatozoa. Abnormalities in this equilibrium lead to spermatogenic failure and male infertility. Here, we introduce PRAMEF12 as an important regulator of spermatogonial biology that is expressed in undifferentiated and early differentiating spermatogonia. We document that genetic ablation of *Pramef12* impairs SSC self-renewal and early differentiation, which prevents sustained cycles of spermatogenesis and results in a Sertoli cell-only syndrome in adult mice. Comparative bulk and scRNA-seq data provide evidence that *Pramef12* deficiency dysregulates previously reported SSC maintenance- and differentiation-related genes and results in impaired germline stem cell development. Comparison with normal transcriptomes of SSCs further demonstrates that *Pramef12* deficiency significantly impairs stem cell potential and blocks stem cell differentiation. Taken together, these data conclusively establish PRAMEF12 as an essential factor in maintenance of the spermatogenic lineage in the testis, which is essential to ensure robust spermatogenesis and male fertility.

Previous studies have identified molecular markers at defined stages of undifferentiated stem and progenitor spermatogonia (Fig. 4j). Except for NANOS2, all are expressed in other tissues and presumably have additional roles in development. Within the testes, expression of ID4, BMI1, PAX7, and EOMES is restricted to a subset of A_s_ spermatogonia^39–41^. GFRA1, UTF1, and NANOS2 are limited to A_s_, A_pr_, and short A_al_ spermatogonia^42–44^, whereas PLZF, SALL4, LIN28A, CDH1, and FOXO1 are expressed later in undifferentiated cells as well as in early differentiated spermatogonia^14,45–50^. Although detected in the placenta, PRAMEF12 is mostly expressed in adult germ cells and genetic ablation has a sterile phenotype in male, but not female, mice. Whole-mount staining of *Pramef12*^*HA/mCherry*^ transgenic testes indicates that PRAMEF12 is co-expressed with PLZF in most, but not all, undifferentiated spermatogonia.

Despite substantial progress in investigating male germline stem cell maintenance, our understanding of the underlying mechanisms that regulate SSC homeostasis remains incomplete. Compared with the reported phenotypes of *Id4*, *Nanos2,* and *Plzf* knockout mice, the defects in *Pramef12*^*Null*^ mice arise much earlier and are more severe, suggesting critical function(s) for PRAMEF12 in regulating early SSC self-renewal and proliferative expansion. However, the continued presence of PRAMEF12 protein in early differentiating spermatogonia suggests that this germ cell-specific protein also regulates spermatogonial differentiation and meiosis. This is supported by the observation that KIT expression, essential for spermatogonial differentiation and initiation of meiosis^51–53^, is significantly decreased in *Pramef12*^*Null*^ testes. This is associated with decreased numbers of meiotic spermatocytes in P14 and P21 tubules and the failure of the first wave of spermatogenesis to result in mature spermatozoa at P35. The experimental observation that transgenic expression of the recombinant protein restores spermatogenesis and fertility of *Pramef12*^*Null*^ mice, confirms that PRAMEF12 drives SSC functions and spermatogenesis in the mouse testes.

Recent studies have reported that PLZF physically interacts with SALL4, and ChIP-seq analysis of PLZF and SALL4 identifies shared regulatory targets (*Bcl6b*, *Etv5*, *Foxo1*, *Lhx1*) involved in SSC self-renewal and differentiation^47,54^. To further elucidate PRAMEF12 function, we performed RNA-seq analysis and found that a series of genes involved in SSC self-renewal (*Eomes*, *Gfra1*, *Sall4*, *Plzf*), differentiation (*Dmrtb1*, *Stra8*, *Kit*, *Sohlh1*, *Sohlh2*) and spermatogenesis (*Piwil2*, *Dazl*, *Ddx4*) were significantly downregulated in *Pramef12*^*Null*^ testes. These observations suggest that cytoplasmic PRAMEF12 participates in gene activation and acts upstream of genes involved in SSC self-renewal and differentiation. However, unlike cytoplasmic NANOS2 that binds RNA^55^, PRAMEF12 does not have RNA-binding motifs and it remains unclear how PRAMEF12 regulates gene expression.

Based on analysis of ID4-positive spermatogonia and expression of other markers in a subset of A_s_ spermatogonia, a hierarchical model has been proposed that supports heterogeneity among SSCs and undifferentiated spermatogonia^9,10^. To profile the transcriptome-wide signatures during germline stem cell development and how PRAMEF12 effects this process, we performed focused re-clustering of *Pramef12*^*Het*^ and *Pramef12*^*Null*^ spermatogonial cells. Based on our scRNA-seq data, we find that: (1) four spermatogonial states (SPG1-4) follow a continuous differentiation trajectory and that *Pramef12* deficiency impairs germline stem cell development as evidenced by decreased percentages of differentiated cells in SPG3/4 states; (2) *Pramef12* deficiency disrupts SSC maintenance- and differentiation-related genes; and (3) the percentage of spermatogonia expressing SSC maintenance-related genes are markedly reduced in *Pramef12*^*Null*^ cells. A recent study reported the developmental trajectory of SSCs and their prospermatogonial precursors by scRNA-seq analysis^38^. To define the SSC fate in the absence of PRAMEF12, we combined the scRNA-seq data on germ cells from P0, P3, and P6 testes with our scRNA-seq data on P7 *Pramef12*^*Het*^ and *Pramef12*^*Null*^ germ cells and compared the transcriptome signatures. These analyses further validate our observations that *Pramef12* deficiency severely impairs stem cell potential and blocks stem cell differentiation.

In summary, our investigations introduce PRAMEF12 as a newly identified cytoplasmic factor that is required for spermatogonial maintenance and is essential for spermatogenesis and fertility. Not only do these findings provide new insights into molecular mechanisms that regulate SSC homeostasis and differentiation, they also provide a better understanding of male germline stem cell biology, which may translate into treatment of human male infertility.

## Methods

### Animals

All animal studies were performed in accordance with guidelines of the Animal Care and Use Committee of the National Institutes of Health under a Division of Intramural Research, NIDDK approved animal study protocol (protocol numbers K018LCDB18 and K044LCDB19).

### Generation of *Pramef12*^*Null*^ mice by CRISPR/Cas9

The guide RNA sequence (5′-GGCTTTGCATTGCGAGCTGT-3′) was designed to target DNA sequence downstream of the *Pramef12* start codon. Double-stranded synthetic DNA was cloned into pDR274 (Addgene, #42250) expressing a single guide RNA (sgRNA). After linearization by digestion with DraI, the DNA fragment was purified with PCR Clean-up Kit (Clontech Laboratories) and transcribed using AmpliScribe T7-Flash Transcription Kit (Lucigen). *Cas9* mRNA (Addgene #42251) was generated by linearization with PmeI, purified with PCR clean-up kit, and transcribed with mMESSAGE mMACHINE T7 Kit (Thermo Fisher Scientific). After transcription, the sgRNA and *Cas9* mRNA were purified with MEGAclear Transcription Clean-Up Kit (Thermo Fisher Scientific). Hormonally stimulated B6D2_F1_ (C57LB/6 × DBA2) female mice were mated to B6D2_F1_ male mice and zygotes were collected from oviducts at embryonic day 0.5 (E0.5). sgRNA (50 ng μl^*−*1^) and *Cas9* mRNA (100 ng μl^*−*1^) were mixed and injected into the zygotes in M2 medium. Injected zygotes were cultured in KSOM (37 °C, 5% CO_2_) to the two-cell embryo stage. Two-cell embryos were transferred into the oviducts of pseudo-pregnant ICR female mice at E0.5. Primers for genotyping the *Pramef12*^*Null*^ mice are listed in Supplementary Table 1. Uncropped gels are presented in Supplementary Fig. 8.

### Generation of transgenic mice

To establish *Pramef12*^*HA/mCherry*^ and *Pramef12*^*FLAG/6xHis/HA*^ transgenic mice, *Pramef12* cDNA with N-terminus HA and C-terminus mCherry or with N-terminus FLAG-6xHis and C-terminus HA were inserted into a pmCherry-N1 vector (Clontech). A 3.2 kb DNA sequence upstream of the transcription start site of *Pramef12* was amplified from mouse genomic DNA and used as a promoter. A DNA fragment including the promoter and the coding sequence was obtained by digestion of the plasmid with NgoMIV. The transgene was microinjected into the male pronucleus of zygotes and embryo transfers were performed as described above. Genotyping primers for *Pramef12*^*HA/mCherry*^ and *Pramef12*^*FLAG/6xHis/HA*^ transgenic mice were designed to bridge the protein-coding and mCherry sequence or the promoter and protein-coding sequence, respectively (Supplementary Table 1).

### Fertility assay

To assess fertility, pairs of *Pramef12*^*Null*^ and *Pramef12*^*Het*^ female mice were co-caged with either a control *Pramef12*^*Het*^ or homozygous *Pramef12*^*Null*^ male mouse for 6 months. The average number of pups per litter was quantified and at least three mating cages were set up for each genotype. A similar approach was used to assess the fertility of *Pramef12*^*Null*^ and *Pramef12*^*Het*^ rescued by the presence of a transgene expressing recombinant PRAMEF12.

### RNA isolation and quantitative real-time RT-PCR

Total RNA was isolated from mouse tissues using RNeasy Mini Kit (Qiagen) and cDNA was synthesized with SuperScript III First-Strand Synthesis System (Thermo Fisher Scientific). Quantitative RT-PCR was performed using iTaq Universal SYBR Green Supermix (Bio-Rad) and QuantStudio 6 Flex Real-Time PCR System (Thermo Fisher Scientific). The primers used in these experiments are listed in Supplementary Table 2. The relative abundance of each transcript was calculated by the 2^−ΔΔ*Ct*^ normalized to endogenous *β-actin* expression^56^. Uncropped gels of Fig. 4a are presented in Supplementary Fig. 8.

### Histology and IF

Mouse testes and epididymides were fixed in Bouin’s solution or 4% paraformaldehyde (PFA) overnight at 4 °C for histology or immunostaining, respectively. Samples were embedded in paraffin, sectioned (5 μm-thick) and mounted on slides prior to staining with periodic acid-Schiff (PAS) and hematoxylin. Cell apoptosis was measured by terminal deoxynucleotidyl transferase-mediated deoxyuridine triphosphate (TUNEL) assay using an In situ Apoptosis Detection Kit (Millipore) according to the manufacturer’s instructions.

After de-waxing, rehydration, and antigen retrieval with 0.01% sodium citrate buffer (pH 6.0) (Sigma Aldrich), tissue sections were blocked with SuperBlock Blocking Buffer containing 0.05% Tween-20 at RT for 1 h and incubated with primary antibodies (Supplementary Table 3) overnight at 4 °C. For IF, secondary antibodies conjugated to Alexa Fluors (Supplementary Table 3) were used to detect the antigen and DNA was stained with Hoechst 33342. For immunohistochemistry, ImmPRESS Polymer Detection Kit (Vector Laboratories) and diaminobenzidine were used to detect antibody binding.

For whole-mount staining, freshly isolated testes were fixed in 4% PFA overnight at 4 °C, permeabilized in SuperBlock Blocking Buffer containing 5% donkey serum, 3% bovine serum albumin, 0.5% Triton X-100 and 0.05% Tween-20 overnight at 4 °C and then incubated with primary antibodies (Supplementary Table 3) for 3–5 days at 4 °C. Secondary antibodies conjugated to Alexa Fluors (Supplementary Table 3) were incubated with the testes for an additional 3–5 days at 4 °C. After washing in PBS, testes were mounted with PBS on the slides. Bright field images were obtained with an inverted microscope (AxioPlan 2; Carl Zeiss) and fluorescent images were captured with a confocal microscope (LSM 780; Carl Zeiss).

### Immunoblot

Total protein was extracted in 1 × LDS sample buffer with 1 × NuPAGE Sample Reducing Agent (Thermo Fisher Scientific). Proteins were separated on 4–12% Bis-Tris gels and electrophoretically transferred to polyvinylidene difluoride membranes. The membranes were blocked with 5% nonfat milk in Tris-buffered saline containing 0.05% Tween-20 (TBST) at RT for 1 h and probed with primary antibodies (Supplementary Table 3) overnight at 4 °C. The membranes then were washed three times with TBST and incubated (1 h, RT) with secondary antibodies (Supplementary Table 3), washed with TBST and developed using SuperSignal West Dura Extended Duration Substrate (Thermo Fisher Scientific). Signals were detected with PXi Touch (SYNGENE) or Hyperfilm ECL (GE Healthcare) according to the manufacturer’s instructions. Uncropped blots are presented in Supplementary Fig. 8.

### Isolation of single testicular cells by FACS

Single cells were isolated according to published protocols^57,58^ with minor modifications. In brief, P7 *Pramef12*^*Null*^ and *Pramef12*^*Het*^ testes were collected and de-capsulated in Hank’s Balanced Salt Solution (HBSS, Gibco). Testicular tubules were digested in 15 ml conical tubes containing 5 ml (1 mg ml^*−*1^) of collagenase (Type IV, Sigma Aldrich)/DNase I (Sigma Aldrich) solution in HBSS at 37 °C with gentle agitation for 15 min. The dispersed tubules were then digested with 0.25% trypsin/EDTA and DNase I at 37 °C with gentle agitation for 7 min. When most of the cells were dispersed, trypsin was neutralized by adding 20% fetal bovine serum (FBS). The cell suspensions were filtered through a pre-wetted 70-μm cell strainer (Corning) and were pelleted by centrifugation at 300 × *g* for 5 min. The cell pellets were resuspended in HBSS with 15% FBS at a concentration of 1 × 10^6^ cells ml^*−*1^.

For FACS analysis, the cells were stained with 1 μg ml^*−*1^ DAPI to exclude dead cells and stained with DRAQ5 dye (Thermo Fisher Scientific) to quantify DNA content, filtered through a 40-μm cell strainer (Corning) before loading on a MoFlo Astrios EQ high speed cell sorter (Beckman Coulter). Flow data analysis was performed using Summit software V6.3.016900 (Beckman Coulter). Sorting strategies for isolation vital single testicular cells were shown in Supplementary Fig. 9. Cells were sorted into HBSS supplemented with 15% FBS and freshly isolated cells were immediately used for scRNA-seq library preparation. FACS experiments were performed at NINDS Flow Cytometry Core Facility in a BSL2 enclosure.

### RNA-seq library preparation

Total RNA was isolated using RNeasy Mini Kit and mRNA was purified by Dynabeads mRNA Purification Kit (Thermo Fisher Scientific). First-strand cDNA was synthesized with SuperScript III Reverse Transcriptase and the second strand was synthesized in 100 μl containing: 20 μl of the first-strand cDNA synthesis mix, 10 μl of 10 × second strand buffer (500 mm Tris-HCl, pH 7.5; 50 mm MgCl_2_; 10 mm DTT), 3 μl of dNTP mix (10 mm), 1 μl of RNaseH (2 U μl^*−*1^), 5 μl of DNA Pol I (10 U μl^*−*1^) and 61 μl H_2_O. The mixture was incubated for 2 h at 16 °C. The libraries were constructed with Nextera DNA Sample Preparation Kit (Illumina) per the manufacturer’s protocol in which double-strand cDNA was fragmented, subjected to adapter ligation and amplified. The final PCR amplified libraries were sequenced at the NIDDK Genomic Core Facility.

### scRNA-seq library preparation

scRNA-seq libraries were prepared using single-cell 3′ reagent kits v2 (10× Genomics) according to the manufacturer’s instructions at the NHLBI Core Facility. In brief, cells obtained from FACS were mixed with a suspension containing barcoded beads and UMI (unique molecular identifier) elements that allow specific tagging of messenger RNA. After partitioning thousands of cells into nanoliter-scale Gel Bead-In-EMulsions (GEMs) and barcoding, full-length barcoded cDNA is then amplified by PCR to generate sufficient mass for library construction. Libraries were constructed by fragmentation, end repair, A-tailing, adaptor ligation, and index PCR. After ensuring adequate quality of the cDNA libraries, the samples were sequenced at the NHLBI Genomic Core Facility.

### RNA-seq analysis

Raw sequence reads were trimmed with cutadapt v1.16 to remove any adapters while performing light quality trimming using parameters -q 20 -a AGATCGGAAGAGC–minimum-length 25. Trimmed reads were mapped to the mm10 reference genome using HISAT v2.1.0 with default parameters^59^ and multimapping reads were filtered using SAMtools v1.7^60^. Uniquely aligned reads were then mapped to gene features using subread featureCounts v1.6.1 with default parameters^61^. Differential expression between groups of samples was tested in R v3.4.1 with DESeq2 v1.18.1^62^.

### scRNA-seq data processing

Raw read processing was carried out using the CellRanger Single-Cell Software Suite (version 3.0.0, 10× Genomics Inc., CA). In brief, the demultiplexed FASTQ files (26 bp Cell barcode and UMI Read1, 8 bp i7 index, and 100 bp Read2) were generated using the CellRanger *mkfastq* command. The primary data analyses that included alignment, filtering, barcode counting and UMI quantification for determining gene transcript counts per cell (generated a gene-barcode matrix), quality control, clustering and statistical analysis were performed using CellRanger *count* command. Gene positions were annotated using Ensembl build 93 and filtered for biotype (only protein-coding, long intergenic non-coding RNA, antisense, immunoglobulin or T-cell receptor).

### Single-cell transcriptomes to identify cell types

Raw gene expression matrices generated per sample using CellRanger (version 3.0.0) were imported into R (version 3.5.0) and converted to a Seurat object using the Seurat R package (version 2.3.4)^63^. Cells that had either fewer than 300 expressed genes, over 30,000 or below 1619 UMIs, or over 15% UMIs derived from mitochondrial genome were discarded. For the remaining 10,060 cells, gene expression matrices were normalized to total cellular read count and to mitochondrial read count using negative binomial regression method implemented in Seurat *RegressOut* function. Cell Cycle scores were also calculated using Seurat *CellCycleScoring* function since the Cell cycle phase effect was observed. The gene expression matrices were then further normalized to cell cycle scores. The variably expressed genes were selected from the normalized data. The gene that had a normalized expression value between 0.13 and 8, a quantile-normalized variance exceeding 0.9, and was not a mitochondrial or ribosome protein-coding genes was defined as highly variably genes (HVG). The resulting 1334 HVGs were used as features for dimensionality reduction and clustering. The Seurat *RunPCA* and *JackStraw* functions was performed to select principal components (PCs), which hold the most differences to separate the cells. The *RunTSNE* function with default setting was then applied to plot the selected significant PCs. We further performed the batch effect correction using Hormany^64^ because a batch effect between two samples was observed. The *RunTSNE* function with default setting was applied to plot the first 25 Harmony aligned coordinates. The *FindClusters* function with resolution = 0.4 parameter was carried out to cluster cells into different groups. The canonical marker genes were applied to annotated cell clusters to known biological cell types.

### Re-clustering of the germ cell types

To identify sub-clusters within spermatogonia-specific cell type, we re-analyzed cells that belonged to spermatogonial cell type separately. Specifically, we selected the HVGs for spermatogonial cell type as described above, and then applied principle component analysis on the selected HVGs for dimensionality reduction. To identify which principle components were informative, we determined statistical significance of PCA scores using Seurat function *JackStraw*, selecting those principle components with *P* value < 0.5. The batch effect correction was also performed as described above. First 20 Harmony aligned coordinates further summarized to UMAP dimensionality reduction using the default of the UMAP function *RunUMAP*^37^. Using the graph-based clustering approach implemented in the *FindClusters* function of the Seurat package, with a conservative resolution of 0.8 and otherwise default parameters, cells were re-clustered by UMAP aligned coordinates.

### Identification of marker genes

To identify marker genes for these nine cell types, we compared the gene expression values of cells from the cluster of interest with that of cells from the rest of clusters using the Seurat *FindMarkers* function with default parameter of Wilcoxon rank-sum test. Marker genes were defined based on the following criteria: (1) the average expression value in the cluster of interest was at least 2.5-fold higher than the average expression in the rest of clusters; (2) there are > 10% of cells in the cluster of interest that were detectable; and (3) marker genes should have the highest mean expression in the cluster of interest compared with the rest of clusters.

### Statistical analysis

Unless otherwise noted, data are presented as the mean ± s.d. The two-tailed Student’s *t* test was used to calculate *Ρ* values. *Ρ* < 0.05 was considered statistically significant.

### Reporting summary

Further information on research design is available in the Nature Research Reporting Summary linked to this article.

## Supplementary information

Supplementary Information

Description of Additional Supplementary Files

Supplementary Data 1

Supplementary Data 2

Supplementary Data 3

Reporting Summary

Source Data

## Data Availability

The accession number for the sequencing data reported in this study has been deposited in the Gene Expression Omnibus website with accession code GSE117708. The source data underlying Figs. 1b, 1d, 1h, 2b, 2e, 2k, 3d, 5d, 6c, and Supplementary Figs. 2c, 2d, 3d, 3f, 4b, 4d, 5b, 5d, 5g, 5k are provided as a Source Data file. A reporting summary for this article is available as a Supplementary Information file. All other relevant data that support the findings of this study are available from the corresponding authors upon reasonable request.

## References

[1] de Rooij DG, Russell LD (2000). All you wanted to know about spermatogonia but were afraid to ask. J. Androl..

[2] Oatley JA, Brinster RL (2008). Regulation of spermatogonial stem cell self-renewal in mammals. Annu Rev. Cell Dev. Biol..

[3] De Felici M (2000). Regulation of primordial germ cell development in the mouse. Int. J. Dev. Biol..

[4] de Rooij DG (2001). Proliferation and differentiation of spermatogonial stem cells. Reproduction.

[5] McLaren A (2000). Germ and somatic cell lineages in the developing gonad. Mol. Cell Endocrinol..

[6] Phillips BT, Gassei K, Orwig KE (2010). Spermatogonial stem cell regulation and spermatogenesis. Philos. Trans. R. Soc. Lond. B Biol. Sci..

[7] de Rooij DG, Grootegoed JA (1998). Spermatogonial stem cells. Curr. Opin. Cell Biol..

[8] Tegelenbosch RA, de Rooij DG (1993). A quantitative study of spermatogonial multiplication and stem cell renewal in the C3H/101 F1 hybrid mouse. Mutat. Res.

[9] Lord T, Oatley JM (2017). A revised A(single) model to explain stem cell dynamics in the mouse male germline. Reproduction.

[10] de Rooij DG (2017). The nature and dynamics of spermatogonial stem cells. Development.

[11] Nakagawa T, Nabeshima Y, Yoshida S (2007). Functional identification of the actual and potential stem cell compartments in mouse spermatogenesis. Dev. Cell.

[12] Nakagawa T, Sharma M, Nabeshima Y, Braun RE, Yoshida S (2010). Functional hierarchy and reversibility within the murine spermatogenic stem cell compartment. Science.

[13] Hara K (2014). Mouse spermatogenic stem cells continually interconvert between equipotent singly isolated and syncytial states. Cell Stem Cell.

[14] Gassei K, Orwig KE (2013). SALL4 expression in gonocytes and spermatogonial clones of postnatal mouse testes. PLoS ONE.

[15] Klein AM, Nakagawa T, Ichikawa R, Yoshida S, Simons BD (2010). Mouse germ line stem cells undergo rapid and stochastic turnover. Cell Stem Cell.

[16] Helsel AR (2017). ID4 levels dictate the stem cell state in mouse spermatogonia. Development.

[17] Chan F (2014). Functional and molecular features of the Id4+ germline stem cell population in mouse testes. Genes Dev..

[18] Soyal SM, Amleh A (2000). Dean J. FIGalpha, a germ cell-specific transcription factor required for ovarian follicle formation. Development.

[19] Joshi S, Davies H, Sims LP, Levy SE, Dean J (2007). Ovarian gene expression in the absence of FIGLA, an oocyte-specific transcription factor. BMC Dev. Biol..

[20] Ikeda H (1997). Characterization of an antigen that is recognized on a melanoma showing partial HLA loss by CTL expressing an NK inhibitory receptor. Immunity.

[21] Chang TC (2011). The expansion of the PRAME gene family in Eutheria. PLoS ONE.

[22] Simpson AJG, Caballero OL, Jungbluth A, Chen YT, Old LJ (2005). Cancer/testis antigens, gametogenesis and cancer. Nat. Rev. Cancer.

[23] Zendman AJW, Ruiter DJ, Van Muijen GNP (2003). Cancer/testis-associated genes: Identification, expression profile, and putative function. J. Cell Physiol..

[24] Enkhbayar P, Kamiya M, Osaki M, Matsumoto T, Matsushima N (2004). Structural principles of leucine-rich repeat (LRR) proteins. Proteins.

[25] Kobe B, Kajava AV (2001). The leucine-rich repeat as a protein recognition motif. Curr. Opin. Struct. Biol..

[26] Mistry BV (2013). Differential expression of PRAMEL1, a cancer/testis antigen, during spermatogenesis in the mouse. PLoS ONE.

[27] Birtle Z, Goodstadt L, Ponting C (2005). Duplication and positive selection among hominin-specific PRAME genes. BMC Genomics.

[28] Wang PJ, McCarrey JR, Yang F, Page DC (2001). An abundance of X-linked genes expressed in spermatogonia. Nat. Genet..

[29] Dade S, Callebaut I, Mermillod P, Monget P (2003). Identification of a new expanding family of genes characterized by atypical LRR domains. Localization of a cluster preferentially expressed in oocyte. FEBS Lett..

[30] Minami N (2003). Oogenesin is a novel mouse protein expressed in oocytes and early cleavage-stage embryos. Biol. Reprod..

[31] Monti M, Redi C (2009). Oogenesis specific genes (Nobox, Oct4, Bmp15, Gdf9, Oogenesin1 and Oogenesin2) are differentially expressed during natural and gonadotropin-induced mouse follicular development. Mol. Reprod. Dev..

[32] Casanova EA (2011). Pramel7 mediates LIF/STAT3-dependent self-renewal in embryonic stem cells. Stem Cells.

[33] Bortvin A (2003). Incomplete reactivation of Oct4-related genes in mouse embryos cloned from somatic nuclei. Development.

[34] Green CD (2018). A comprehensive roadmap of murine spermatogenesis defined by single-cell RNA-seq. Dev. Cell.

[35] Hobbs RM, Seandel M, Falciatori I, Rafii S, Pandolfi PP (2010). Plzf regulates germline progenitor self-renewal by opposing mTORC1. Cell.

[36] Suzuki H (2012). SOHLH1 and SOHLH2 coordinate spermatogonial differentiation. Dev. Biol..

[37] Becht Etienne, McInnes Leland, Healy John, Dutertre Charles-Antoine, Kwok Immanuel W H, Ng Lai Guan, Ginhoux Florent, Newell Evan W (2018). Dimensionality reduction for visualizing single-cell data using UMAP. Nature Biotechnology.

[38] Law NC, Oatley MJ, Oatley JM (2019). Developmental kinetics and transcriptome dynamics of stem cell specification in the spermatogenic lineage. Nat. Commun..

[39] Oatley MJ, Kaucher AV, Racicot KE, Oatley JM (2011). Inhibitor of DNA binding 4 is expressed selectively by single spermatogonia in the male germline and regulates the self-renewal of spermatogonial stem cells in mice. Biol. Reprod..

[40] Aloisio GM (2014). PAX7 expression defines germline stem cells in the adult testis. J. Clin. Invest.

[41] Komai Y., et al. Bmi1 expression in long-term germ stem cells. *Sci. Rep.* **4**, 6175 (2014). 10.1038/srep06175 PMC4141270 25146451

[42] Suzuki H, Sada A, Yoshida S, Saga Y (2009). The heterogeneity of spermatogonia is revealed by their topology and expression of marker proteins including the germ cell-specific proteins Nanos2 and Nanos3. Dev. Biol..

[43] van Bragt MP (2008). Expression of the pluripotency marker UTF1 is restricted to a subpopulation of early A spermatogonia in rat testis. Reproduction.

[44] Meng XJ (2000). Regulation of cell fate decision of undifferentiated spermatogonia by GDNF. Science.

[45] Buaas FW (2004). Plzf is required in adult male germ cells for stem cell self-renewal. Nat. Genet.

[46] Costoya JA (2004). Essential role of Plzf in maintenance of spermatogonial stem cells. Nat. Genet.

[47] Hobbs RM (2012). Functional antagonism between Sall4 and Plzf defines germline progenitors. Cell Stem Cell.

[48] Tokuda M, Kadokawa Y, Kurahashi H, Marunouchi T (2007). CDH1 is a specific marker for undifferentiated spermatogonia in mouse testes. Biol. Reprod..

[49] Goertz MJ, Wu ZR, Gallardo TD, Hamra FK, Castrillon DH (2011). Foxo1 is required in mouse spermatogonial stem cells for their maintenance and the initiation of spermatogenesis. J. Clin. Invest.

[50] Fayomi AP, Orwig KE (2018). Spermatogonial stem cells and spermatogenesis in mice, monkeys and men. Stem Cell Res..

[51] Zhang L (2013). c-kit expression profile and regulatory factors during spermatogonial stem cell differentiation. BMC Dev. Biol..

[52] Busada JT (2015). Retinoic acid regulates Kit translation during spermatogonial differentiation in the mouse. Dev. Biol..

[53] Manku G, Culty M (2015). Mammalian gonocyte and spermatogonia differentiation: recent advances and remaining challenges. Reproduction.

[54] Lovelace DL (2016). The regulatory repertoire of PLZF and SALL4 in undifferentiated spermatogonia. Development.

[55] Sada A, Suzuki A, Suzuki H, Saga Y (2009). The RNA-binding protein NANOS2 is required to maintain murine spermatogonial stem cells. Science.

[56] Livak KJ, Schmittgen TD (2001). Analysis of relative gene expression data using real-time quantitative PCR and the 2(T)(-Delta Delta C) method. Methods.

[57] Guan K (2009). Isolation and cultivation of stem cells from adult mouse testes. Nat. Protoc..

[58] Valli H (2014). Fluorescence- and magnetic-activated cell sorting strategies to isolate and enrich human spermatogonial stem cells. Fertil. Steril..

[59] Kim D, Landmead B, Salzberg SL (2015). HISAT: a fast spliced aligner with low memory requirements. Nat. Methods.

[60] Li H (2009). The sequence alignment/map format and SAMtools. Bioinformatics.

[61] Liao Y, Smyth GK, Shi W (2013). The Subread aligner: fast, accurate and scalable read mapping by seed-and-vote. Nucleic Acids Res..

[62] Love MI, Huber W, Anders S (2014). Moderated estimation of fold change and dispersion for RNA-seq data with DESeq2. Genome Biol..

[63] Butler A, Hoffman P, Smibert P, Papalexi E, Satija R (2018). Integrating single-cell transcriptomic data across different conditions, technologies, and species. Nat. Biotechnol..

[64] Korsunsky, I. et al. Fast, sensitive, and accurate integration of single cell data with Harmony. Preprint at *bioRxiv* 10.1101/461954 (2018). 10.1038/s41592-019-0619-0 PMC6884693 31740819

