# Supplementary material for: Sertoli cell-only phenotype and scRNA-seq define PRAMEF12 as a factor essential for spermatogenesis in mice
Source: Nat Commun. 2019 Nov 15;10:5196. doi: 10.1038/s41467-019-13193-3 (PMC6858368; doi:10.1038/s41467-019-13193-3)
Supplement: Supplementary file 1 — Supplementary Information [file 41467_2019_13193_MOESM1_ESM.pdf]

Supplementary information

Sertoli cell-only phenotype and scRNA-seq define PRAMEF12 as a factor essential for spermatogenesis in mice

Wang *et al*.

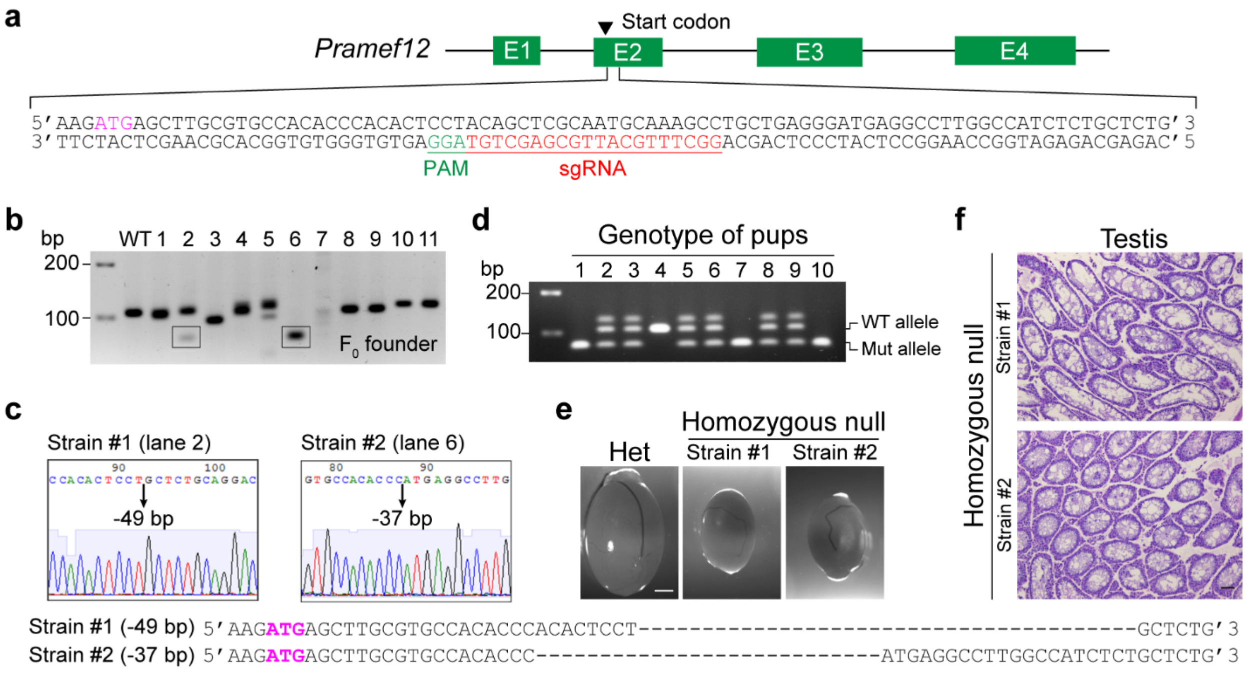

Supplementary Figure 1 Generation of *Pramef12* null mice. **a** Exon map of the mouse *Pramef12* locus based on Ensembl (ENSMUSG00000028591). Exons, green; ATG start codon, purple; AGG, the PAM (protospacer adjacent motif) sequence, green; guide RNA, red 20-nt sequence. **b** Two *Pramef12*^{Null} founders (lanes 2, 6) with **c** deletions (-49 bp and -37 bp) were confirmed by DNA sequence. **d** PCR genotyping to detect WT, heterozygous and homozygous *Pramef12*^{Null} mice. Third band is a heteroduplex. **e** Testes from adult mice of the indicated genotypes. Scale bar, 1 mm. **f** PAS staining of the testicular sections from homozygous strain #1 and strain #2 adult *Pramef12*^{Null} mice. Scale bar, 50 µm. Representative of n=3 (**e**, **f**) independent biological replicates with similar results per condition.

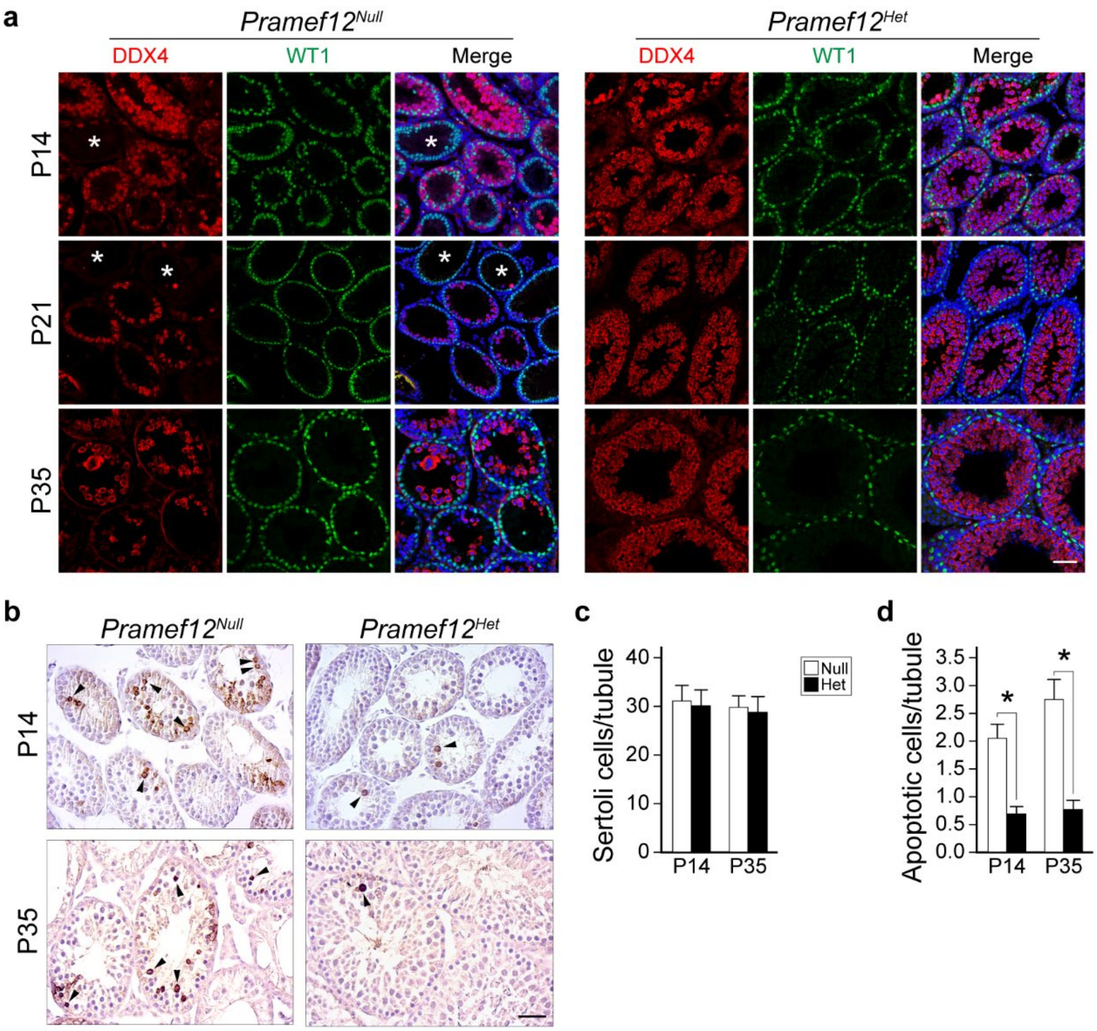

Supplementary Figure 2 Germ cell loss and apoptosis in *Pramef12*^{Null} mice. **a** Immunofluorescence of testes sections from P14, P21 and P35 *Pramef12*^{Null} and *Pramef12*^{Het} mice after staining with antibodies to DDX4 (left) or WT1 (middle) and merged with Hoechst 33342 to detect DNA (right). The average number of DDX4-positive germ cells per tubular cross-section was significantly reduced in the *Pramef12*^{Null} testes. Asterisks, Sertoli cell-only tubules. Scale bar, 50 μm. **b** Cellular apoptosis was analyzed with TUNEL assay in P14 and P35 *Pramef12*^{Null} and *Pramef12*^{Het} testes. Arrowheads, apoptotic germ cells. Scale bar, 50 μm. **c** Average number of Sertoli cells per tubule was quantified in P14 and P35 *Pramef12*^{Null} and *Pramef12*^{Het} testes. Mean ± s.d, n=3 biologically independent samples at each age point. **d** The

number of apoptotic cells per tubule was analyzed from P14 and P35 *Pramef12*^{Null} and *Pramef12*^{Het} testes. Mean ± s.d, n=3 biologically independent samples at each age point. **P* =2.91E-04 (P14) and **P* =4.71E-10 (P35) by two-tailed Student's t test. Representative of n=3 (**a**, **b**) independent biological replicates with similar results per condition.

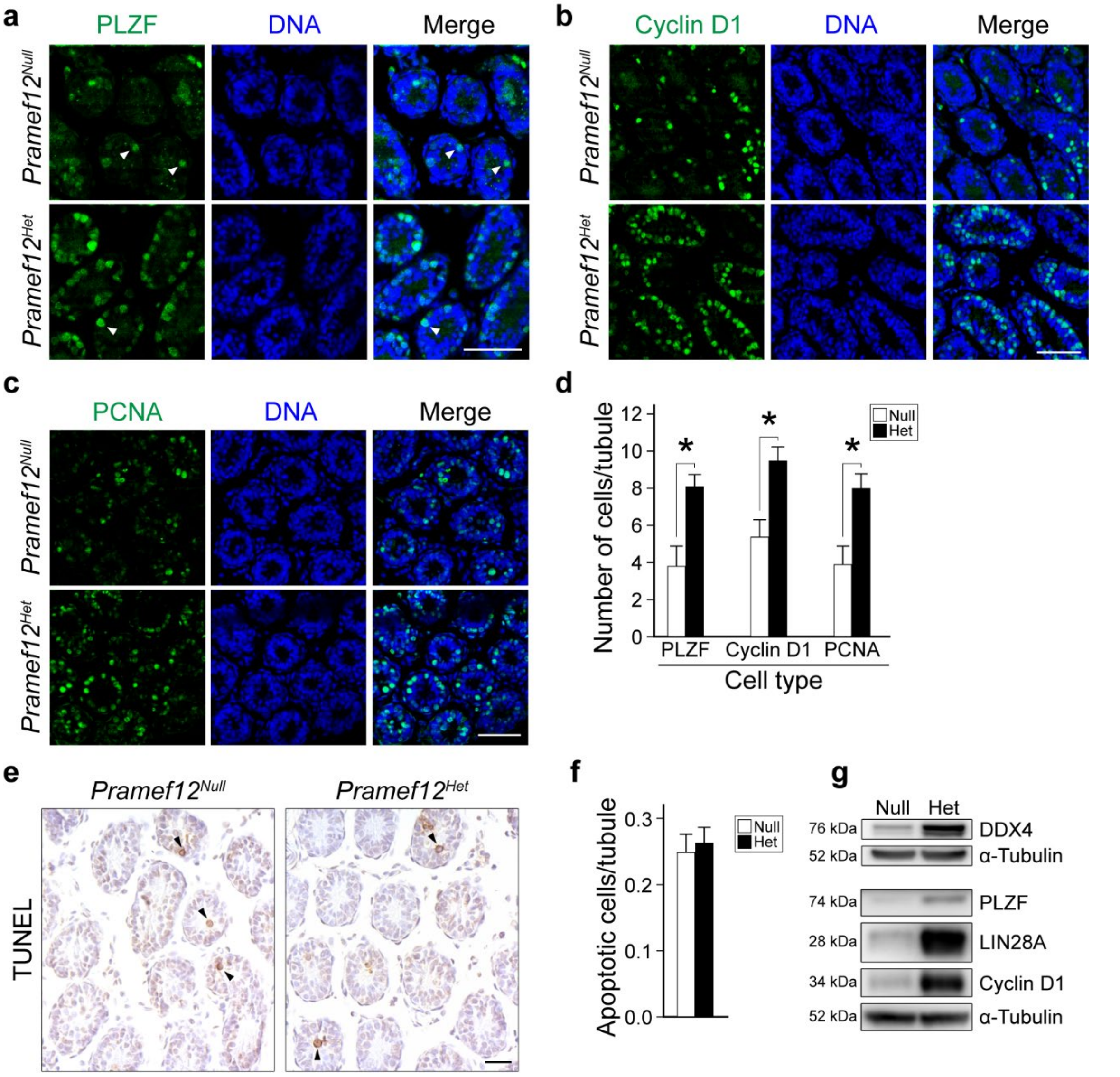

Supplementary Figure 3 Proliferation and apoptosis of spermatogonia. **a** Immunofluorescence of testes sections from P7 *Pramef12*^{Null} and *Pramef12*^{Het} mice after staining with antibodies to PLZF (left) or with Hoechst 33342 to stain DNA (middle) and merged (right). Arrowheads, PLZF-positive spermatogonia. Scale bar, 50 μm. **b** Same as **a**, but with antibodies to cyclin D1 (marker of mitosis). **c** Same as **a**, but with antibodies to PCNA (marker of proliferation). **d** Comparison of the number of PLZF-, cyclin D1- and PCNA-positive cells per seminiferous tubules of P7 *Pramef12*^{Null} and *Pramef12*^{Het} testes. Mean ± s.d, n=3 biologically independent samples. **P* =2.20E-12 (PLZF), **P* =3.33E-11 (Cyclin D1) and **P* =7.14E-09 (PCNA) by two-

tailed Student's t test. **e** Cellular apoptosis was analyzed by TUNEL assay in P7 *Pramef12*^{Null} and *Pramef12*^{Het} testes. Arrowheads, apoptotic germ cells. Scale bar, 50 μm. **f** Number of apoptotic cells per tubule of P7 *Pramef12*^{Null} and *Pramef12*^{Het} testes. Mean ± s.d, n=3 biologically independent samples. **g** Immunoblot of DDX4, PLZF, Lin28a and cyclin D1 in P7 *Pramef12*^{Null} and *Pramef12*^{Het} testes using α-tubulin as a load control. Representative of n=3 (**a**-**c**, **e**, **g**) independent biological replicates with similar results per condition.

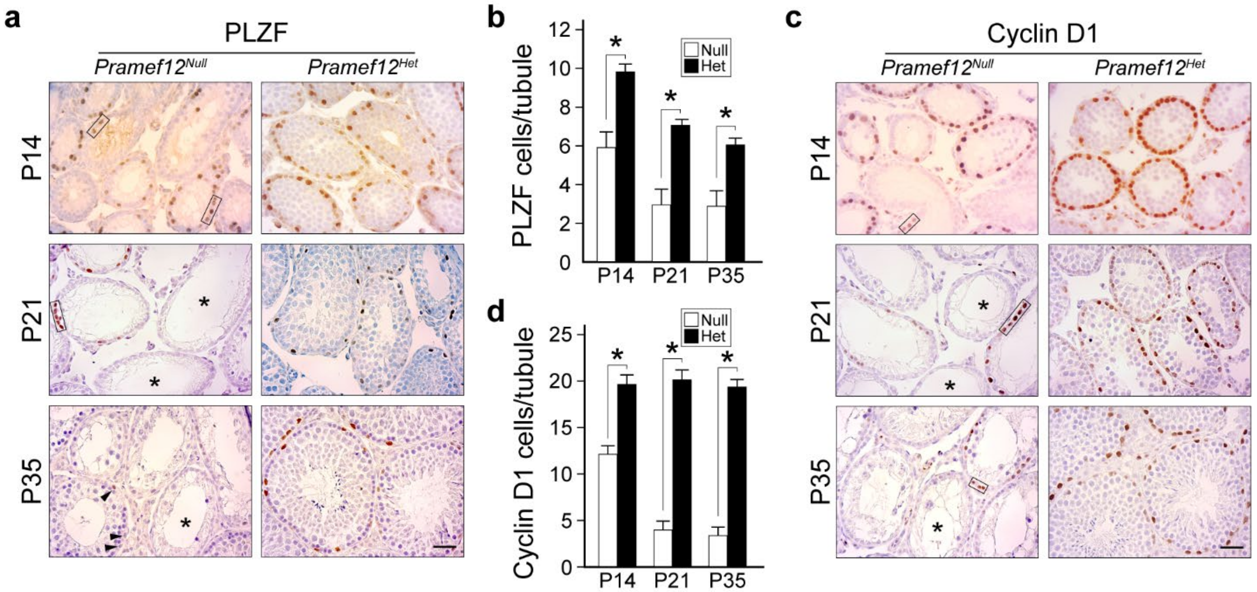

Supplementary Figure 4 Expression patterns of PLZF and cyclin D1. **a** Immunohistochemistry of P14, P21 and P35 testes from *Pramef12^{Null}* and *Pramef12^{Het}* mice after staining with antibodies to PLZF and counterstaining with hematoxylin. Arrowheads, PLZF-positive spermatogonia; rectangles, PLZF-positive spermatogonia at the basement membrane; asterisks, agametic tubules. Scale bar, 50 μm. **b** Quantitative comparison of PLZF-positive spermatogonia per tubule from P14, P21 and P35 *Pramef12^{Null}* and *Pramef12^{Het}* mice. Mean ± s.d, n=3 biologically independent samples at each age point. *P =2.17E-17 (P14), *P =2.97E-13 (P21) and *P =1.55E-13 (P35) by two-tailed Student's t test. **c** Same as **a**, but with antibodies to cyclin D1. Rectangles, cyclin D1-positive spermatogonia at the basement membrane; asterisks, agametic tubules. **d** Same as **b**, but for cyclin D1-positive spermatogonia. Mean ± s.d, n=3 biologically independent samples at each age point. *P =3.68E-08 (P14), *P =1.24E-18 (P21) and *P =7.20E-23 (P35) by two-tailed Student's t test. Representative of n=3 (**a**, **c**) independent biological replicates with similar results per condition.

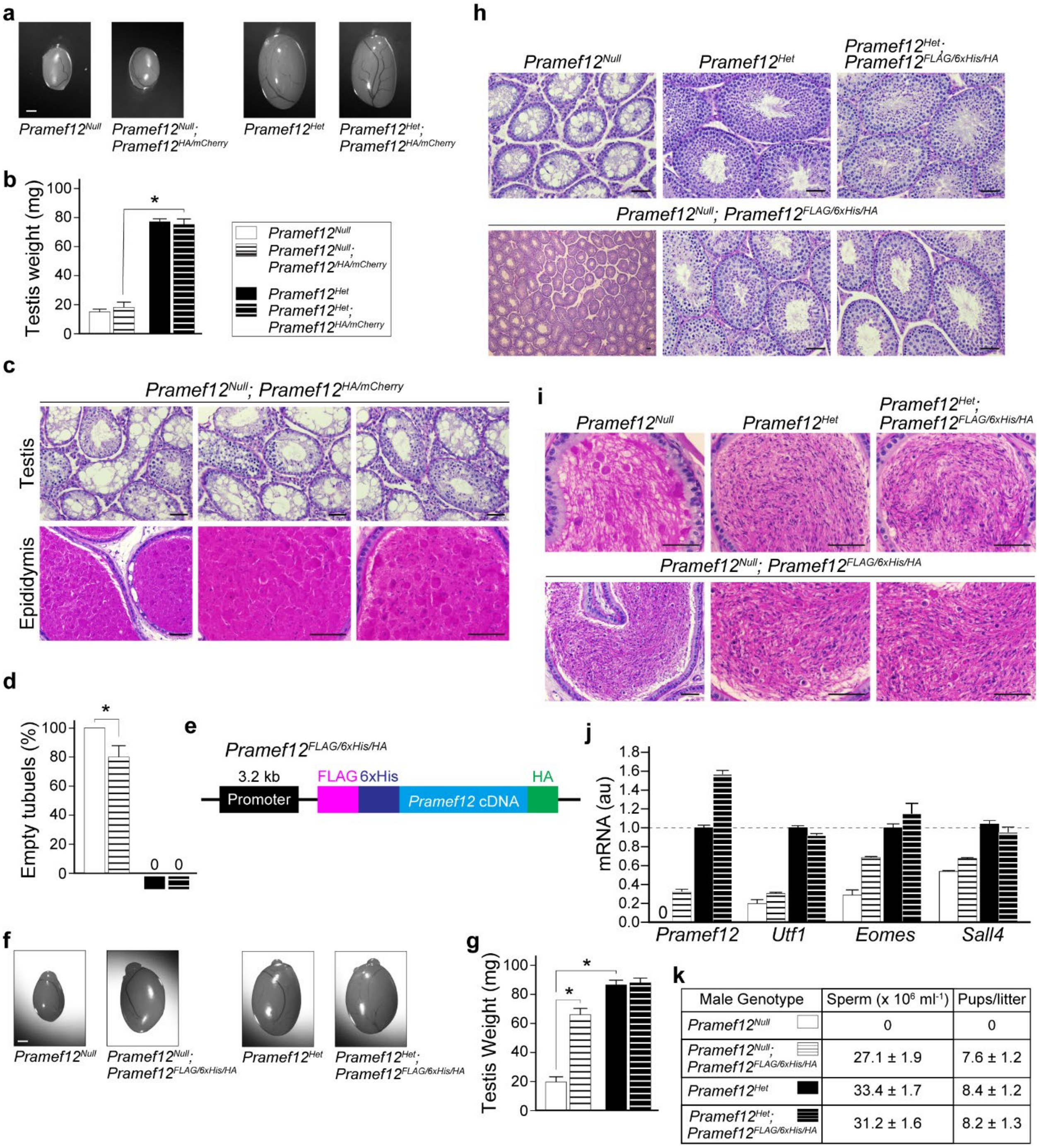

Male Genotype	Sperm (x 10^6 ml^{-1})	Pups/litter
Pramef12Null	0	0
Pramef12Null; Pramef12FLAG/6xHis/HA	27.1 ± 1.9	7.6 ± 1.2
Pramef12Het	33.4 ± 1.7	8.4 ± 1.2
Pramef12Het; Pramef12FLAG/6xHis/HA	31.2 ± 1.6	8.2 ± 1.3

Supplementary Figure 5 Transgenic expression of *Pramef12* restores spermatogenesis and fertility of *Pramef12*^{Null} mice. **a** Testes from adult P90 *Pramef12*^{Null}, *Pramef12*^{Null}; *Pramef12*$^{HA/mCherry}$; *Pramef12*^{Het} and *Pramef12*^{Het}; *Pramef12*$^{HA/mCherry}$ mice. Scale bar, 1 mm. **b** Comparison of testes weight from (**a**). Mean ± s.d, n=3 biologically independent samples. *P

=1.15E-10 by two-tailed Student's t test. **c** Adult testis (top panels) and epididymis (bottom panels) sections from *Pramef12*^{Null}; *Pramef12*$^{HA/mCherry}$ mice stained with PAS and hematoxylin. Scale bar, 50 μm. **d** Percentage of agametic tubules in adult *Pramef12*^{Null}, *Pramef12*^{Null}; *Pramef12*$^{HA/mCherry}$; *Pramef12*^{Het} and *Pramef12*^{Het}; *Pramef12*$^{HA/mCherry}$ mice. Around 20% of tubules contain spermatogenic cells in *Pramef12*^{Null}; *Pramef12*$^{HA/mCherry}$ testes. Graph legend in (**b**). Mean ± s.d, n=3 biologically independent samples. **P* =2.96E-07 by two-tailed Student's t test. **e** Schematic representation of transgene expressing $^{FLAG/6xHis}$*Pramef12*^{HA} cDNA driven by the *Pramef12* promoter (3.2 kb). **f** Testes of P90 adult *Pramef12*^{Null}, *Pramef12*^{Null}; *Pramef12*$^{FLAG/6xHis/HA}$; *Pramef12*^{Het} and *Pramef12*^{Het}; *Pramef12*$^{FLAG/6xHis/HA}$ mice. Scale bar, 1 mm. **g** Comparison of testes weight from (**f**). Graph legend in (**b**). Mean ± s.d, n=3 biologically independent samples. **P* =2.15E-07 (low) and **P* =8.14E-09 (high) by two-tailed Student's t test. **h** Adult testis sections from *Pramef12*^{Null}, *Pramef12*^{Het}, *Pramef12*^{Het}; *Pramef12*$^{FLAG/6xHis/HA}$ (top) and *Pramef12*^{Null}; *Pramef12*$^{FLAG/6xHis/HA}$ (bottom) mice stained with PAS and hematoxylin. Transgenic expression of *Pramef12* rescued spermatogenesis in *Pramef12*^{Null} tubules. Scale bar, 50 μm. **i** Same as (**h**), but of cauda epididymides. **j** RNA abundance of *Pramef12*, *Utf1*, *Eomes* and *Sall4* from the P7 testes with indicated genotypes. Graph legend in (**b**). Mean ± s.d, n=3 biologically independent replicates. **k** Sperm concentration and litter size (3 mating pairs) with indicated genotypes in which *Pramef12*^{Null}; *Pramef12*$^{FLAG/6xHis/HA}$ males exhibited similar sperm concentration and litter sizes as *Pramef12*^{Het} and *Pramef12*^{Het}; *Pramef12*$^{FLAG/6xHis/HA}$ control mice. Data are presented as mean ± s.d for n=3 biologically independent replicates. Representative of n=3 (**a**, **c**, **f**, **h**, **i**) independent biological replicates with similar results per condition.

a

Summary for each sample (cleaned data)

Samples	Total cell	Total UMI	Total genes	Mean genes/cell	Median genes/cell	Mean UMI/cell	Median UMI/cell
Pramef12^{Null}	4,839	35,373,902	20,561	2,417	2,153	7,310	6,172
Pramef12^{Het}	5,221	39,529,914	21,027	2,481	2,092	7,571	5,846
Total	10,060	74,903,816	21,961	2,450	2,126	7,446	6,009

b

Summary of cell number for each cluster

Cluster/cell type	*Pramef12*^{Het} (cells)	Total Het cell (%)	*Pramef12*^{Null} (cells)	Total Null cell (%)
Unknown 1	1,126	21.6	1,204	24.9
Unknown 2	875	16.8	1,043	21.6
Spermatogonia	1,151	22.1	656	13.6
Sertoli	802	15.4	767	15.9
Myoid	415	8.0	326	6.7
Leydig	243	4.7	416	8.6
Cell cycle-related	367	7.0	186	3.8
Macrophage	212	4.1	200	4.1
Myoid 2	20	0.4	31	0.6
Endothelial	10	0.2	10	0.2

c

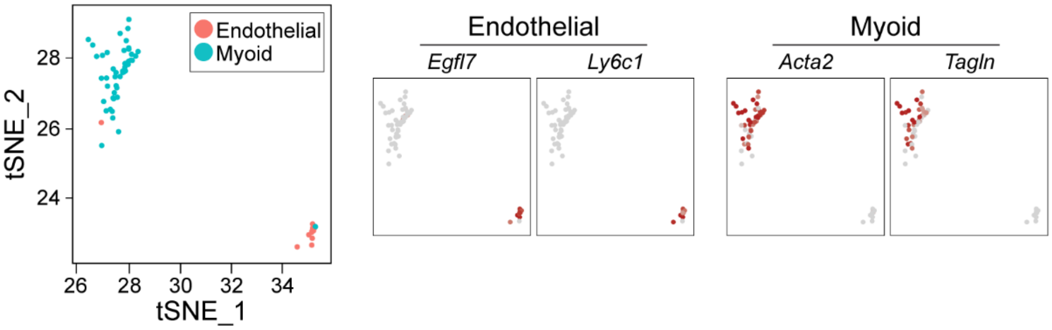

d

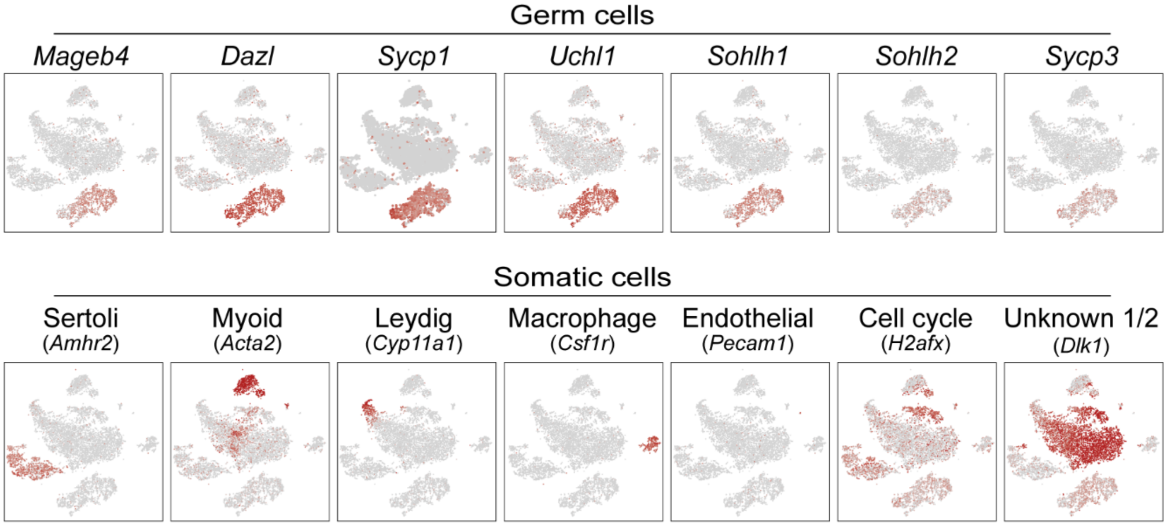

Supplementary Figure 6 scRNA-seq profiles of P7 *Pramef12*^{Het} and *Pramef12*^{Null} testicular cells and cell type identification. **a** Sequencing metrics after quality control of scRNA-seq datasets. **b** Specific cell numbers of *Pramef12*^{Het} and *Pramef12*^{Null} testicular cells in each cluster. **c** Focused re-clustering of myoid 2 cluster cells and selected marker genes of myoid and endothelial cells on tSNE plots. **d** Gene expression patterns of additional marker genes in germ cells (top) and somatic cells (bottom) projected onto the tSNE plots.

a

Summary of cell number in subtypes within cluster 2 (Spermatogonia)

Cluster Subtype	*Pramef12*^{Het} (cells)	*Pramef12*^{Het} (% subtype)	*Pramef12*^{Het} (% total)	*Pramef12*^{Null} (cells)	*Pramef12*^{Null} (% subtype)	*Pramef12*^{Null} (% total)
SPG1	255	22.2	4.9	233	35.5	4.8
SPG2	128	11.0	2.4	196	29.9	4.1
SPG3	514	44.7	9.8	107	16.3	2.2
SPG4	256	22.2	4.9	120	18.3	2.5
Total	1171	NA	22.1	656	NA	13.6

b

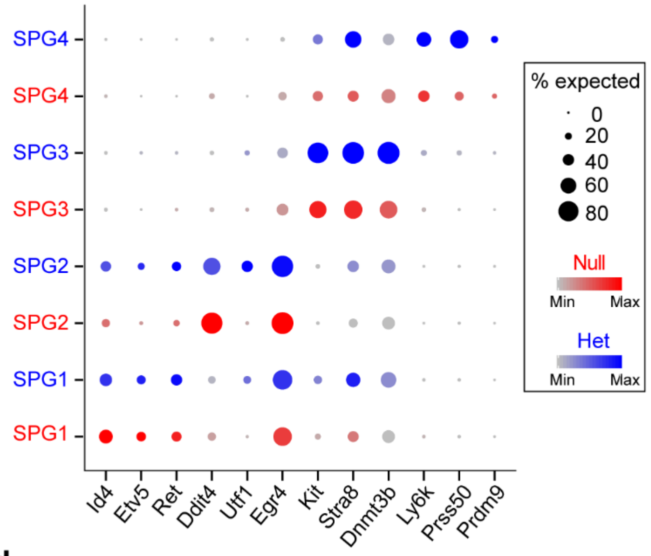

c

	Enriched GO terms	*P* value
SPG1	Regulation of metabolic process	1.68E-08
	Regulation of apoptotic process	1.65E-07
	Regulation of gene expression	1.74E-07
	Positive regulation of biological process	3.32E-07
	Cell proliferation	4.55E-07
SPG2	Translation	1.01E-26
	Peptide biosynthetic process	9.20E-26
	Peptide metabolic process	2.42E-24
	Amide biosynthetic process	7.88E-23
	rRNA processing	1.00E-09
SPG3	Negative regulation of cellular process	1.41E-07
	Negative regulation of biological process	3.98E-07
	Negative regulation of metabolic process	1.25E-06
	Regulation of gene expression	1.68E-04
	Regulation of cell proliferation	9.15E-04
SPG4	Cellular metabolic process	8.38E-20
	Cell cycle	6.03E-14
	Cellular biosynthetic process	2.52E-09
	Cell division	8.97E-08
	Meiotic cell cycle process	1.15E-05

d

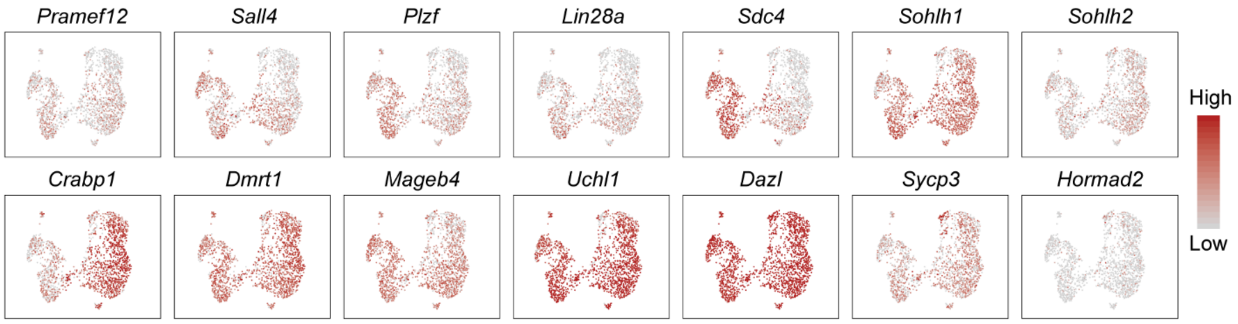

e

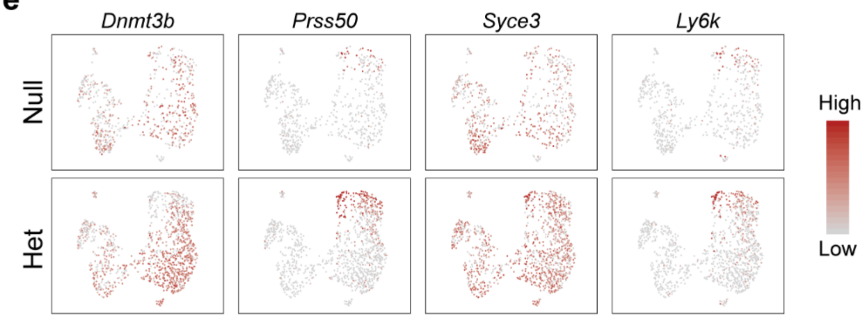

Supplementary Figure 7 Identification of four subtypes of spermatogonia and DEGs in each subtype. **a** Summary of specific cell numbers of spermatogonia in each cluster in *Pramef12*^{Het} and *Pramef12*^{Null} testes. **b** Dot plot for expression of selected marker genes across the four SPG subtypes. **c** Enriched GO terms (top) and *P* values of the four SPG subtypes were obtained by GOrilla analysis. *P* values were directly obtained from the data by GOrilla analysis. **d** Gene expression patterns of selected spermatogonial marker genes visualized in UMAP plots. *Pramef12*, *Sall4*, *Plzf*, *Lin28a* and *Sdc4* transcripts showing similar expression patterns in spermatogonia, were mainly expressed in SPG1, SPG2 and SPG3 cell types which correspond to undifferentiated and early differentiating spermatogonia. **e** UMAP plots of the expression patterns of selected DEGs in SPG3 and SPG4 in *Pramef12*^{Het} and *Pramef12*^{Null} samples.

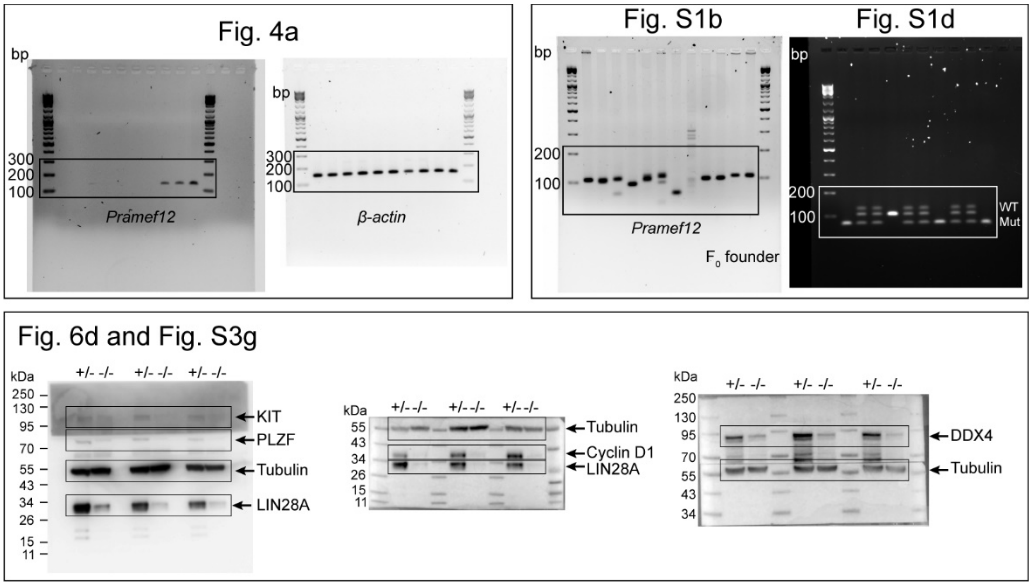

Supplementary Figure 8 Uncropped images of PCR gels and blots. Rectangles indicate areas that were cropped for use in figures.

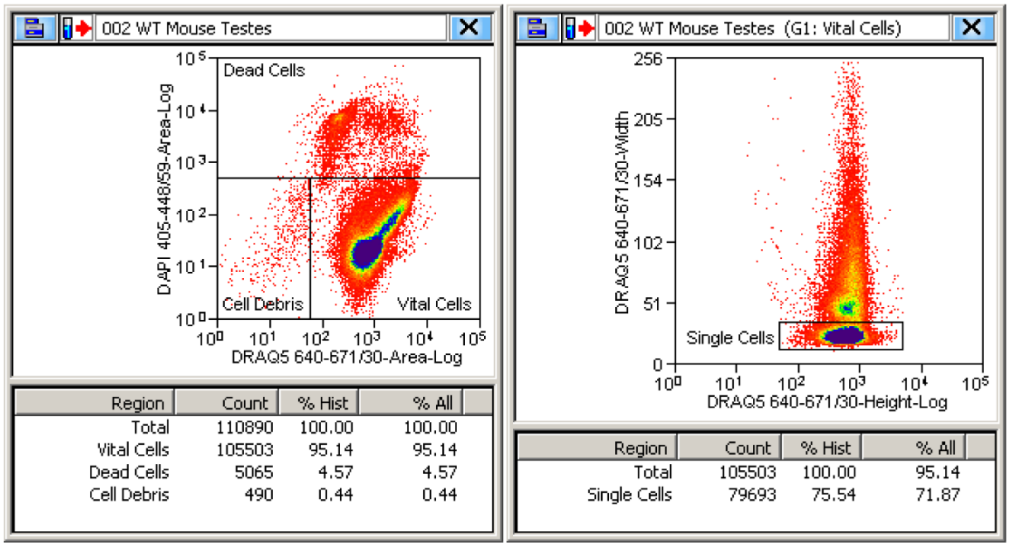

Supplementary Figure 9 Sorting strategies for isolation vital single testicular cells. FACS plots exemplify the gating strategy that we used based on DAPI and DRAQ5 staining to exclude dead cells and debris and to sort only vital single cells based on DRAQ5 signal height versus width pulse processing.

Supplementary Table 1 Genotyping primers for *Pramef12* null and transgenic mice.

Gene	Direction	Primer (5'-3')
Pramef12^{Null}	F[1]	CCTTGGGCATTTTCTCAGGAT
	R	TCCATGGGCAGGTCCTGCAGA
Pramef12$^{HA/mCherry}$	F	AACAGAACGGTCTGGTTCAGCG
	R	AAGGACAGCTTCAAGTAGTCGG
Pramef12$^{FLAG/6xHis/HA}$	F	AATAACCCACGACACCAGGAC
	R	CGCCCACAGGAAGACATAGGA

[1]F, forward; R, reverse

Supplementary Table 2 Primers used for RT-PCR in this study.

Gene	Direction	Primers (5'-3')
Pramef12	F[1]	TACAGCTCGCAATGCAAAGC
	R	CCTCAGGATGTTAAGTCGTTTGT
Utf1	F	TGTCCCGGTGACTACGTCT
	R	CCCAGAAGTAGCTCCGTCTCT
Eomes	F	GCGCATGTTTCCTTTCTTGAG
	R	GGTCGGCCAGAACCACTTC
Pou5f1	F	AGAGGATCACCTTGGGGTACA
	R	CGAAGCGACAGATGGTGGTC
Lin28a	F	TGGTGTGTTCTGTATTGGGAGT
	R	AGTTGTAGCACCTGTCTCCTTT
Sall4	F	CCCTGGGAACTGCGATGAAG
	R	TCAGAGAGACTAAAGAACTCGGC
Gfra1	F	CACTCCTGGATTTGCTGATGT
	R	AGTGTGCGGTACTTGGTGC
Ret	F	TTTCTCAAGGGATGCTTACTGGG
	R	CCCGTAGGGCATGGACATAGA
Plzf	F	CTGGGACTTTGTGCGATGTG
	R	CGGTGGAAGAGGATCTCAAACA
Dmrtb1	F	AATCCTGAGAGAGAGCCGTTC
	R	GCCGGAAGCCTCTTTGTTGA
Stra8	F	CAAAAGCCTTGGCTGTGTTA
	R	AAAGGTCTCCAGGCACTTCA
Kit	F	GCCTGACGTGCATTGATCC
	R	AGTGGCCTCGGCTTTTTCC
Sohlh1	F	CGGGCCAATGAGGATTACAGA
	R	TCCTGCGTTCTCTCTCGCT
Lin28b	F	GCCTTGAGTCAATACGGGTAAC
	R	AGGGTCTTCCCTTTAGGTCTTC
Rarg	F	AAGTACACCACGAACTCCAGT
	R	TTCGCAAACTCCACAATCTTGA
Dnmt3b	F	AGCGGGTATGAGGAGTGCAT
	R	GGGAGCATCCTTCGTGTCTG
Sohlh2	F	GGGCAGGGCAGAGTAAATCTT
	R	CAAACGAGTTAGCAGCCAAAAG
Piwil2	F	TTGGCCTCAAGCTCCTAGAC
	R	GAACATGGACACCAAACCTACA
Dazl	F	ATGTCTGCCACAACTTCTGAG
	R	CTGATTTCGGTTTCATCCATCCT
Ddx4	F	GAAGAAATCCAGAGGTTGGC
	R	GAAGGATCGTCTGCTGAACA
Btbd18	F	GTTCCTCCGGGTAGCTTTTCT
	R	GCTCCAAGCGTTCTGTGAAG

Tdrd1	F	TCTTCAACTCGTTAGGACCGC
	R	CCGTGGAGCAGTAGTACGTCT
β-actin	F	GGCTGTATTCCCCTCCATCG
	R	CCAGTTGGTAACAATGCCATGT

[1]F, forward; R, reverse

Supplementary Table 3 Antibodies used for immunohistochemistry and immunoblot.

Antibody	Company	Identifier	Immuno-blot	Immunohisto-chemistry
Rabbit anti-DDX4	Abcam	Cat# ab13840; RRID: AB_443012	1:1000	1:200
Mouse anti-DDX4	Abcam	Cat# ab27591; RRID: AB_11139638		1:200
Rabbit anti-WT1	Abcam	Cat# ab89901; RRID: AB_2043201		1:200
Rabbit anti-mCherry	Abcam	Cat#ab167453; RRID: AB_2571870		1:300
Rabbit anti-Cyclin D1	Abcam	Cat# ab134175; RRID: AB_2750906	1:1000	1:200
Goat anti-PLZF	R&D Systems	Cat# AF2944; RRID: AB_2218943	1:200	1:200
Goat anti-KIT	R&D Systems	Cat# AF1356; RRID: AB_354750	1:200	1:200
Goat anti-GFRA1	R&D Systems	Cat# AF560; RRID: AB_2110307		1:200
Rabbit anti-KI67	Cell Signaling Technology	Cat# 9129; RRID: AB_2687446		1:200
Rabbit anti-Phospho-Histone H2A.X (Ser139)	Cell Signaling Technology	Cat# 9718; RRID: AB_2118009		1:200
Rabbit anti-LIN28A	Cell Signaling Technology	Cat# 8641S; RRID: AB_10997528	1:1000	
Mouse anti-PCNA	Santa Cruz	Cat# sc-56; RRID: AB_628110		1:500
Mouse anti-alpha Tubulin	Thermo Fisher Scientific	Cat# 62204; RRID: AB_1965960	1:1000	
Donkey anti-Mouse IgG, Alexa Fluor 488	Thermo Fisher Scientific	Cat# A-21202		1:200
Donkey anti-Rabbit IgG, Alexa Fluor 594	Thermo Fisher Scientific	Cat# A-21207		1:200
Donkey anti-Goat IgG, Alexa Fluor 488	Thermo Fisher Scientific	Cat# A-11055		1:200
Donkey anti-Goat IgG, Alexa Fluor 633	Thermo Fisher Scientific	Cat# A-21082		1:200
Goat anti-Mouse IgG, HRP	Thermo Fisher Scientific	Cat# 62-6520	1:5000	

Goat anti-Rabbit IgG, HRP	Thermo Fisher Scientific	Cat# 31460	1:5000	
Donkey anti-Goat IgG, HRP	Thermo Fisher Scientific	Cat# PA1-28664	1:5000	
