# Supplementary material for: Sertoli cell-only phenotype and scRNA-seq define PRAMEF12 as a factor essential for spermatogenesis in mice
Source: Nat Commun. 2019 Nov 15;10:5196. doi: 10.1038/s41467-019-13193-3 (PMC6858368; doi:10.1038/s41467-019-13193-3)
Supplement: Supplementary file 2 — Description of Additional Supplementary Files [file 41467_2019_13193_MOESM2_ESM.pdf]

# Description of Additional Supplementary Files

File Name: Supplementary Data 1

Description: Identification of cell types in juvenile testes. Combined P7 Pramef12Het and Pramef12Null testicular cells were divided into 9 subtypes according to marker genes. Differentially expressed genes were shown in each cluster.

File Name: Supplementary Data 2

Description: Identification of spermatogonial states. Re-clustering of P7 Pramef12Het and Pramef12Null spermatogonial cells. Spermatogonia were divided into 4 subtypes. Differentially expressed genes were shown for each subtype.

File Name: Supplementary Data 3

Description: Transcriptome differences between Pramef12Null and Pramef12Het cells. Differentially expressed genes between Pramef12Null and Pramef12Het cells were shown for each subtype.
