# Supplementary material for: Sertoli cell-only phenotype and scRNA-seq define PRAMEF12 as a factor essential for spermatogenesis in mice
Source: Nat Commun. 2019 Nov 15;10:5196. doi: 10.1038/s41467-019-13193-3 (PMC6858368; doi:10.1038/s41467-019-13193-3)
Supplement: Supplementary file 6 — Reporting Summary [file 41467_2019_13193_MOESM6_ESM.pdf]

nature research

Corresponding author(s): Zhengpin Wang, Jurrien Dean

Last updated by author(s): Oct 8, 2019

Reporting Summary

Nature Research wishes to improve the reproducibility of the work that we publish. This form provides structure for consistency and transparency in reporting. For further information on Nature Research policies, see Authors & Referees and the Editorial Policy Checklist.

Statistics

For all statistical analyses, confirm that the following items are present in the figure legend, table legend, main text, or Methods section.

n/a	Confirmed	
☐	☒	The exact sample size (*n*) for each experimental group/condition, given as a discrete number and unit of measurement
☐	☒	A statement on whether measurements were taken from distinct samples or whether the same sample was measured repeatedly
☐	☒	The statistical test(s) used AND whether they are one- or two-sided<br>*Only common tests should be described solely by name; describe more complex techniques in the Methods section.*
☒	☐	A description of all covariates tested
☐	☒	A description of any assumptions or corrections, such as tests of normality and adjustment for multiple comparisons
☐	☒	A full description of the statistical parameters including central tendency (e.g. means) or other basic estimates (e.g. regression coefficient) AND variation (e.g. standard deviation) or associated estimates of uncertainty (e.g. confidence intervals)
☐	☒	For null hypothesis testing, the test statistic (e.g. *F*, *t*, *r*) with confidence intervals, effect sizes, degrees of freedom and *P* value noted<br>*Give P values as exact values whenever suitable.*
☒	☐	For Bayesian analysis, information on the choice of priors and Markov chain Monte Carlo settings
☒	☐	For hierarchical and complex designs, identification of the appropriate level for tests and full reporting of outcomes
☒	☐	Estimates of effect sizes (e.g. Cohen's *d*, Pearson's *r*), indicating how they were calculated

Our web collection on statistics for biologists contains articles on many of the points above.

Software and code

Policy information about availability of computer code

Data collection	The qRT-PCR data: iTaq Universal SYBR Green Supermix (Bio-Rad) and QuantStudio 6 Flex Real-Time PCR System (Thermo Fisher Scientific).<br>The immunoblot data: PXi Touch (SYNGENE) or Hyperfilm ECL (GE Healthcare).<br>The flow cytometric data: MoFlo Astrios EQ high speed cell sorter (Beckman Coulter).<br>The Image data: Bright field images were obtained with an inverted microscope (AxioPlan 2; Carl Zeiss) and fluorescent images were captured with a confocal microscope (LSM 780; Carl Zeiss).
Data analysis	Statistical analysis: Sigmaplot software (v 12.0).<br>Image data analysis: ImageJ (V1.51t, NIH) and Adobe Photoshop CC 2019.<br>qRT-PCR analysis: The relative abundance of each transcript was calculated by the 2−ΔΔCt normalized to endogenous actinb expression.<br>Flow data analysis: Summit software V6.3.016900 (Beckman Coulter).<br>RNA-seq analysis: Raw sequence reads were trimmed with cutadapt v1.16 to remove any adapters while performing light quality trimming using parameters "-q 20 -a AGATCGGAAGAGC --minimum-length 25". Trimmed reads were mapped to the mm10 reference genome using HISAT v2.1.0 with default parameters and multimapping reads were filtered using SAMtools v1.7. Uniquely aligned reads were then mapped to gene features using subread featureCounts v1.6.1 with default parameters. Differential expression between groups of samples was tested in R v3.4.1 with DESeq2 v1.18.1.<br>scRNA-seq analysis: Raw read processing was carried out using the Cell Ranger Single-Cell Software Suite (version 3.0.0, 10X Genomics Inc., CA). Briefly, the demultiplexed FASTQ files (26bp Cell barcode and UMI Read1, 8bp i7 index, and 100bp Read2) were generated using the CellRanger mkfastq command. The primary data analyses which included alignment, filtering, barcode counting and UMI quantification for determining gene transcript counts per cell (generated a gene-barcode matrix), quality control, clustering and statistical analysis were performed using CellRanger count command. Gene positions were annotated using Ensembl build 93 and filtered for biotype (only protein-coding, long intergenic non-coding RNA, antisense, immunoglobulin or T-cell receptor).<br>Gene Ontology analysis: Gene Ontology enRIchment anaLysis and visuaLizAtion tool (GORILLA) software.

For manuscripts utilizing custom algorithms or software that are central to the research but not yet described in published literature, software must be made available to editors/reviewers. We strongly encourage code deposition in a community repository (e.g. GitHub). See the Nature Research guidelines for submitting code & software for further information.

Data

Policy information about availability of data

All manuscripts must include a data availability statement. This statement should provide the following information, where applicable:

- Accession codes, unique identifiers, or web links for publicly available datasets
- A list of figures that have associated raw data
- A description of any restrictions on data availability

The accession number for the sequencing data reported in this study has been deposited in the Gene Expression Omnibus website with accession code GSE117708. This data is publicly available upon publication of the manuscript.

Field-specific reporting

Please select the one below that is the best fit for your research. If you are not sure, read the appropriate sections before making your selection.

☒ Life sciences ☐ Behavioural & social sciences ☐ Ecological, evolutionary & environmental sciences

For a reference copy of the document with all sections, see nature.com/documents/nr-reporting-summary-flat.pdf

Life sciences study design

All studies must disclose on these points even when the disclosure is negative.

Sample size	Each sample or experimental/biological replicate (n) represented one animal or a single testis from one animal (as noted in the text). At least n=3 biological samples/replicates were collected/performed for each experiment, which is sufficient for statistical analysis.
Data exclusions	No data were excluded.
Replication	Experimental measurements were replicated at least three times. Reproducibility was ensured by each replicate per condition.
Randomization	Mice were randomly allocated to experimental groups respective of their genotype.
Blinding	Blinding was not necessary for this study because the null mutation displayed a distinct phenotype.

Reporting for specific materials, systems and methods

We require information from authors about some types of materials, experimental systems and methods used in many studies. Here, indicate whether each material, system or method listed is relevant to your study. If you are not sure if a list item applies to your research, read the appropriate section before selecting a response.

Materials & experimental systems

n/a	Involved in the study	
☐	☒	Antibodies
☒	☐	Eukaryotic cell lines
☒	☐	Palaeontology
☐	☒	Animals and other organisms
☒	☐	Human research participants
☒	☐	Clinical data

Methods

n/a	Involved in the study	
☒	☐	ChIP-seq
☐	☒	Flow cytometry
☒	☐	MRI-based neuroimaging

Antibodies

Antibodies used	Rabbit anti-DDX4 (Cat# ab13840; RRID: AB_443012), Mouse anti-DDX4 (Cat# ab27591; RRID: AB_11139638), Rabbit anti-WT1 (Cat# ab89901; RRID: AB_2043201), Rabbit anti-mCherry (Cat#ab167453; RRID: AB_2571870), Rabbit anti-Cyclin D1 (Cat# ab134175; RRID: AB_2750906) were purchased from Abcam. Goat anti-PLZF (Cat# AF2944; RRID: AB_2218943), Goat anti-KIT (Cat# AF1356; RRID: AB_354750), Goat anti-GFRA1 (Cat# AF560; RRID: AB_2110307) were purchased from R&D Systems. Rabbit anti-KI67 (Cat# 9129; RRID: AB_2687446), Rabbit anti-Phospho-Histone H2A.X (Ser139) (Cat# 9718; RRID: AB_2118009), Rabbit anti-LIN28A (Cat# 8641S; RRID: AB_10997528) were purchased from Cell Signaling Technology. Mouse anti-PCNA (Cat# sc-56; RRID: AB_628110) was purchased from Santa Cruz. Mouse anti-alpha Tubulin (Cat# 62204; RRID: AB_1965960), Donkey anti-Mouse IgG, Alexa Fluor 488 (Cat# A-21202), Donkey anti-Rabbit IgG, Alexa Fluor 594 (Cat# A-21207), Donkey anti-Goat IgG, Alexa Fluor 488 (Cat# A-11055), Donkey anti-Goat IgG, Alexa Fluor 633 (Cat# A-21082), Goat anti-Mouse IgG, HRP (Cat# 62-6520), Goat anti-Rabbit IgG, HRP (Cat# 31460), Donkey anti-Goat IgG, HRP (Cat# PA1-28664) were purchased from Thermo Fisher Scientific.
Validation	Commercial antibodies were validated by the manufacturers and the statements could be found on the manufactures' websites.

Animals and other organisms

Policy information about studies involving animals; ARRIVE guidelines recommended for reporting animal research

Laboratory animals	Mice used in this study were a mixed B6D2F1 (C57LB/6 × DBA2) background. Postnatal day 2 (P2), P7, P10, P14, P21, P35, P48, P60, P75, P90 Pramef12 heterozygous (Pramef12+/-) and Pramef12 null (Pramef12 -/-) male mice, and adult (2-3 months old) Pramef12HA/mCherry and Pramef12FLAG/6xHis/HA transgenic male mice were used in the experiments.
Wild animals	No wild animals were used.
Field-collected samples	No field-collected samples were used.
Ethics oversight	All animal studies were performed in accordance with guidelines of the Animal Care and Use Committee of the National Institutes of Health under a Division of Intramural Research, NIDDK approved animal study protocol.

Note that full information on the approval of the study protocol must also be provided in the manuscript.

Flow Cytometry

Plots

Confirm that:

☒ The axis labels state the marker and fluorochrome used (e.g. CD4-FITC).

☒ The axis scales are clearly visible. Include numbers along axes only for bottom left plot of group (a 'group' is an analysis of identical markers).

☒ All plots are contour plots with outliers or pseudocolor plots.

☒ A numerical value for number of cells or percentage (with statistics) is provided.

Methodology

Sample preparation	Single cells were isolated according to published protocols with minor modifications. Briefly, P7 Pramef12Null and Pramef12Het testes were collected and de-capsulated in Hank's Balanced Salt Solution (HBSS, Gibco). Testicular tubules were digested in 15 ml conical tube containing 5 ml (1 mg ml-1) of collagenase (Type IV, Sigma Aldrich)/DNase I (Sigma Aldrich) solution in HBSS at 37 °C with gentle agitation for 15 min. The dispersed tubules were then digested with 0.25% trypsin/EDTA and DNase I at 37 °C with gentle agitation for 7 min. When most of the cells were dispersed, trypsin was neutralized by adding 20% fetal bovine serum (FBS). The cell suspensions were filtered through a pre-wetted 70 um cell strainer (Corning) and were pelleted by centrifugation at 300 x g for 5 min. The cell pellets were resuspended in HBSS with 15% FBS at a concentration of 1×106 cells ml-1.
Instrument	MoFlo Astrios EQ high speed cell sorter (Beckman Coulter).
Software	Summit software V6.3.016900 (Beckman Coulter).
Cell population abundance	1×106 live and single cells were obtained from control and mutant testicular cells, respectively.
Gating strategy	Cells were stained with 1 ug ml-1 DAPI to exclude dead cells and stained with DRAQ5 dye (Thermo Fisher Scientific) to quantify DNA content.

☒ Tick this box to confirm that a figure exemplifying the gating strategy is provided in the Supplementary Information.
